# Controlled synchronization of three co-rotating exciters based on a circular distribution in a vibratory system

**DOI:** 10.1038/s41598-024-55680-8

**Published:** 2024-02-29

**Authors:** Lei Jia, Yang Tian, Ziliang Liu, Xin Zhang

**Affiliations:** 1 School of Mechanical Engineering, Shenyang Ligong University, Shenyang, 110159 China; 2 School of Mechanical Engineering, Liaoning Engineering Vocational College, Tieling, 112008 China

**Keywords:** Self-synchronization, Controlled synchronization, Coupling dynamical model, Vibratory system, Exciter rotors, Mechanical engineering, Nonlinear phenomena

## Abstract

In this article, an engineering problem of three co-rotating exciters with the circular distribution in a vibrating system is investigated. The dynamical model constructed by the motion differential equations is established. By introducing the small parameter averaged method in the dynamic equation, the synchronization and stability conditions of the electromechanical coupling dynamical model is derived. To illustrate the necessity of the controlling method, the self-synchronization of the vibrating system is firstly analyzed with the theory, numerical simulations and experiments. With the self-synchronization results, it is indicated that the ellipse trajectory which is needed in the industry can’t be realizefd by the self-synchronization motion of the vibrating system. And then, a fuzzy PID controlling method based on the master–slave controlling strategy is introduced in the vibrating system to realize the controlled synchronization. The Lyapunov stability criterion is given to certify the stability of the controlling system. Through some simulations and experiments, the effectiveness of controlled synchronization is illustrated in the discussion. Finally, the present work illuminates the feasibility and practicality for designing some new types of vibrating screens in the industry.

## Introduction

Nowadays, the vibratory machinery has a rapid development with an improvement of the technology in the industry, for example the vibrating screen, vibrating feeder and so on^1–3^. The vibrating screen is used in various of industry fields because of its large potential economic benefits. The actuators of the vibrating screen with traditional forms usually adopt the forced synchronization motion by gears, belts, chains or some other transmission mechanisms. However, the more mechanism parts are used, the lower reliability the vibrating system is. This result could decrease the useful life of the vibrating system. With the development of the synchronous theory, some synchronous methods are presented by the dynamical intrinsic character. The theory of vibration synchronization is firstly represented by Blekhman et al.^4,5^ In their work, two eccentric rotors (ERs) separately driven by two motors are installed on a vibrating bench with the same frequency. They divide the motion of the vibrating system into two processes with different time scales. One is the faster process and the other is lower process. With this method, they obtain the synchronization and stability conditions of two motors rather than the coupling characteristics of the vibrating system. Inoue et al.^6^ realizes the self-synchronization motion of two and three times frequency with four motors. Wen et al.^7^ have research on the self-synchronization motion with two motors in a vibrating system. In the research, the synchronization and stability conditions of the whole vibrating system is derived with the average method and Hamilton principle. Zhao et al.^8,9^ proposes the small parameter average method based on the perturbation method. And with this method, they substitute the problem of synchronization for existence and stability of the eigenvalues which are obtained from the dynamical equations of the vibrating system. Zhang et al.^10–12^ not only establishes the dynamical model of three motors in a vibrating system, but also provides the experiment results of the self-synchronization. In the meanwhile, the double and triple frequency synchronization is studied. In their research work, the synchronous and stability conditions of the vibrating system with three ERs are derived and the results show that when the synchronous and stability conditions are not satisfied, the circular motion which is needed in the vibratory screen can’t be realized. Balthazar et al.^13,14^ analyze the problem of self-synchronization with four direct current (DC) motors. They use the elastic support to replace the rigid body and introduce the numerical method into the dynamical model instead of the analytical solution.

As illustrated above, the self-synchronization motion should satisfy the synchronous and stability conditions so that it can realize the needed trajectory in the vibratory screen. Aim at this problem of the self-synchronization, the method of controlled synchronization which introduces the controlling method and controlling strategy into the self-synchronization is presented. Kong et al.^15–17^ realizes the controlled synchronization motion of three and four ERs in a vibrating system. The master–slave controlling strategy is used in the controlled system with the adaptive slide controlling method. Besides that, the synchronization based on an elastic beam is also studied. In their work, the approximate circular trajectory is realized with three ERs driven by inductor motors which are installed in one line on a rigid frame. And then, the linear trajectory is given with four ERs driven by inductor motors which are symmetrically installed along the horizontal and vertical axes. Perez-Pinal et al.^18^ represents a controlling strategy titled relative coupling control. Huang et al.^19,20^ introduces the relative coupling controlling method into a non-linear vibrating system and realizes the controlled synchronization motion with two ERs. The multifrequency synchronization with controlling method is studied. Priyanka et al.^21^ uses the fuzzy PID controlling method to realize the fluid machinery control, which provides a way for the controlled synchronization. Jia et al.^22,23^ use the controlled synchronization and composite synchronization method on the multifrequency synchronization motion. In their research, the non-integer multifrequency controlled synchronization is investigated.

Compared with the finished research work, it can be known that the self-synchronization with three ERs can’t realize the approximate circular trajectory which can be realized by controlled synchronization method. However, the ERs driven by inductor motors with the linear distribution need a larger frame. This result may not be suit for some smaller vibratory screen. Thus, an engineering problem of three co-rotating exciters with the circular distribution in a vibrating system is investigated. The structures of present work are as follows: the electromechanical coupling dynamic model of the vibrating system is established in Section "Synchronization and stability analysis of dynamical differential equation". And then the motion differential equation of the dynamic model is derived. The synchronous and stability conditions of the self-synchronization with three ERs is obtained. In Section "Design and theoretical analysis of the controlling system", the controlling method is introduced to establish the controlling system and the stability of the controlling system is certified by the Lyapunov criterion. Section "Results analysis and discussions" shows the discussions with theory, numerical simulations and experiments. Finally, some conclusions are summarized in Section "Conclusions".

## Synchronization and stability analysis of dynamical differential equation

### The establishment of the theoretical model

**Figure 1:**
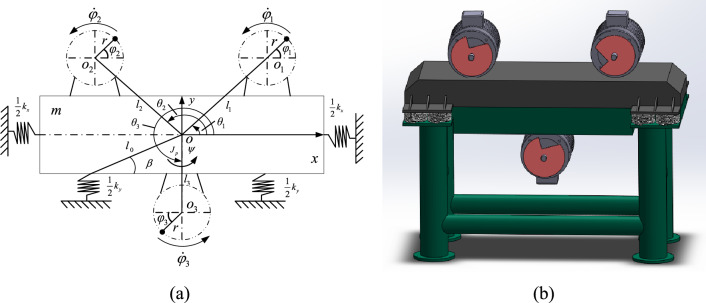
Mathematical model of the vibrating system.

The model of the vibratory screen can be simplified as the theoretical model of a vibrating system shown in Fig. 1. And the significance of mathematical symbols in this paper are all listed in Table 1. The vibrating system is constructed from top to the bottom. Two inductor motors with ERs are symmetrically installed on a rigid frame along the vertical axis. One motor is installed under the rigid frame on the vertical axis. *o*_*i*_(*i* = 1,2,3) are respectively the rotating center of three ERs. The frame is supported by four springs which provide the stiffness in the *x* and *y* directions. *o* is the center of the rigid frame. *M* is the total mass of the vibrating system, $M = m + m_{i} (i = 1,2,3)$. *m* is the mass of the frame and $oo_{i} = l_{i} (i = 1,2,3)$. *J* and *J*_*p*_ are respectively the inertia moment of the vibrating system and the rigid frame, $J = Ml_{e}^{2} \approx J_{p} + \sum\limits_{i = 1}^{3} {m_{i} } (l_{i}^{2} + r^{2} )$. *l*_*e*_ is equivalent rotation radius. *f*_*x*_, *f*_*y*_ and *f*_*ψ*_ are the damping coefficients, $f_{\psi } = l_{0}^{2} (f_{x} \sin^{2} \beta + f_{y} \cos^{2} \beta )$. *k*_*x*_, *k*_*y*_ and *k*_*ψ*_ are the stiffnesses, $k_{\psi } = l_{0}^{2} (k_{x} \sin^{2} \beta + k_{y} \cos^{2} \beta )$. *f*_*i*_ (*i* = 1,2,3) respectively represents damping coefficients of the inductor motor. The parameters of the vibrating system are given in Table 2.

**Table 1:** The nomenclature table of the symbols.

Symbol	Significance
*m*_*i*_	The mass of each ERs
*J*_*p*_	The moment of inertia of the rigid frame
*J*	The moment of inertia of the vibrating system
*k*_*x*_, *k*_*y*_, *k*_*ψ*_	The stiffness coefficients of the vibration system in the *x*, *y* and *ψ* directions
*r*	The eccentric radius of the inductor motors
*M*	The total mass of the vibration system
*f*_*x*_, *f*_*y*_, *f*_*ψ*_	The damping coefficients of the vibration system in the *x*, *y* and *ψ* directions
*l*_1_,* l*_2_,* l*_3_	The distance between the center of the body and the rotating center of motors
*J*_*i*_	The moment of inertia of the inductor motor
*d-*, *q-*	The *d*- and *q*- axes in rotor field-oriented coordinate
*L*_*s*_	Self-inductance of the stator
*L*_*r*_	Self-inductance of the rotor
Subscript *s*	Stator
Subscript* r*	Rotor
*L*_*m*_	Mutual inductance of the stator and rotor
*L*_*ks*_	Leakage inductance of the stator
$\phi_{sd}$	The flux linkages of the stator in the *d*- axis
$\phi_{sq}$	The flux linkages of the stator in the *q*- axis
$\phi_{rd}$	The flux linkages of the rotor in the *d*- axis
$\phi_{rq}$	The flux linkages of the rotor in the *q*- axis
*R*_*s*_	The stator resistance
*R*_*r*_	The rotor resistance
*R*_*ks*_	Equivalent resistance of the stator
*i*_*sd*_	The current of stator in the *d*- axis
*i*_*sq*_	The current of stator in the *q*- axis
*i*_*rd*_	The current of rotor in the *d*- axis
*i*_*rq*_	The current of rotor in the *q*- axis
*ω*	The mechanical speed
*ω*_*s*_	The synchronous electric angular speed
$\dot{\phi }_{sd}$,$\dot{\phi }_{sq}$,$\dot{\phi }_{rd}$,$\dot{\phi }_{rq}$	Derivation of$\phi_{sd}$,$\phi_{sq}$,$\phi_{rd}$,$\phi_{rq}$
*σ*	The leakage factor
*Tr*	A rotor time constant
*u*_*sd*_	The voltage of stator in the *d*- axis
*u*_*sq*_	The voltage of stator in the *q*- axis
*u*_*rd*_	The voltage of rotor in the *d*- axis
*u*_*rq*_	The voltage of rotor in the *q*- axis
*n*_*p*_	The number of pole-pairs of the induction motor
$\bullet^{ * }$	The given values or obtained from the given values
$\omega_{1}$,$\omega_{2}$,$\omega_{3}$	The speeds of three motors
$\varphi_{1}$,$\varphi_{2}$,$\varphi_{3}$	The phases of three motors
${\uptheta }$	Synchronization electric angle

**Table 2:** The parameters of the vibrating system.

Parameters	Values
*M* /kg	304
*J*_*p*_ /(kg.m^2^)	44.5
*k*_*x*_ /(N/m)	129,332
*k*_*y*_ /(N/m)	105,334
*k*_*ψ*_ /(Nm/rad)	30,715
*f*_*x*_ /(Ns/m)	615.5
*f*_*y*_ /(Ns/m)	618
*f*_*ψ*_ /(Nsm/rad)	180.2
*θ*_1_, *θ*_2_, *θ*_3_ /(°)	30, 150, 270
*m*_0_ /kg	6
*r* /m	0.05
*l*_1_, *l*_2_,* l*_3_ /m	0.45, 0.45, 0.36

According to Fig. 1, the mathematical model of the vibrating system can be derived with Lagrange equation.

1 $$\frac{{\text{d}}}{{{\text{d}}t}}\left( {\frac{\partial (T - V)}{{\partial {\dot{\mathbf{q}}}}}} \right) - \frac{\partial (T - V)}{{\partial {\mathbf{q}}}} + \frac{\partial D}{{\partial {\dot{\mathbf{q}}}}} = {\mathbf{Q}}$$

Equation 1 contains four variables, the first is the kinetic energy *T* which is composed of three parts. They are respectively the item of translational energy of the rigid body $T_{teb} = m(\dot{x}^{2} + \dot{y}^{2} )/2$, rotational energy of the rigid body $T_{reb} = J_{b} \dot{\psi }^{2} /2$ and the kinetic energy of three ERs $T_{kee} = \left( {\sum\limits_{i = 1}^{3} {m_{i} } \dot{\delta }_{i}^{{\text{T}}} \dot{\delta }_{i} + \sum\limits_{i = 1}^{3} {J_{i} } \dot{\varphi }_{i}^{2} } \right)/2$, where, $\delta_{i} = \delta_{0} + \chi \delta^{\prime\prime}_{i}$. According to the transformation of the coordinate system, it can be derived as $$\delta_{0} = \left( \begin{gathered} x \hfill \\ y \hfill \\ \end{gathered} \right)$$, $$\chi = \left( \begin{gathered} \cos \psi {\kern 1pt} {\kern 1pt} {\kern 1pt} {\kern 1pt} {\kern 1pt} {\kern 1pt} - \sin \psi \hfill \\ \sin \psi {\kern 1pt} {\kern 1pt} {\kern 1pt} {\kern 1pt} {\kern 1pt} {\kern 1pt} {\kern 1pt} {\kern 1pt} {\kern 1pt} {\kern 1pt} {\kern 1pt} {\kern 1pt} {\kern 1pt} {\kern 1pt} {\kern 1pt} \cos \psi \hfill \\ \end{gathered} \right)$$, $$\delta^{\prime\prime}_{i} = \left( \begin{gathered} l_{i} \cos \theta_{i} + r\cos \varphi_{i} \hfill \\ l_{i} \sin \theta_{i} + r\sin \varphi_{i} \hfill \\ \end{gathered} \right)$$. Thus, the total kinetic energy of the system can be expressed as $T = T_{teb} + T_{reb} + T_{kee}$. The second is the potential energy *V* which can be expressed as $V = (k_{x} x^{2} + k_{y} y^{2} + k_{\psi } \psi^{2} )/2$. The third is the energy dissipation *D* described as $D = \left( {f_{x} \dot{x}^{2} + f_{y} \dot{y}^{2} + f_{\psi } \dot{\psi }^{2} + \sum\limits_{i = 1}^{3} {f_{i} \dot{\varphi }_{i}^{2} } } \right)/2$. The last items are the generalized force ${\mathbf{Q}} = (Q_{x} ,Q_{y} ,Q_{\psi } ,Q_{1} ,Q_{2} ,Q_{3} )^{{\text{T}}} = (0,0,0,T_{e1} ,T_{e2} ,T_{e3} )^{{\text{T}}}$ and the generalized coordinate ${\mathbf{q}} = (x,y,\psi ,\varphi_{1} ,\varphi_{2} ,\varphi_{3} )^{{\text{T}}}$. Through Eq. (1), the differential equations of the electromechanical coupling dynamical model can be expressed as Eq. (2) with the Lagrange function.

2 $$\begin{gathered} M\ddot{x} + f_{x} \dot{x} + k_{x} x = \sum\limits_{i = 1}^{3} {m_{i} } r\left( {\dot{\varphi }_{i}^{2} \cos \varphi_{i} + \ddot{\varphi }_{i} \sin \varphi_{i} } \right) \hfill \\ M\ddot{y} + f_{y} \dot{y} + k_{y} y = \sum\limits_{i = 1}^{3} {m_{i} } r\left( {\dot{\varphi }_{i}^{2} \sin \varphi_{i} - \ddot{\varphi }_{i} \cos \varphi_{i} } \right) \hfill \\ J\ddot{\psi } + f_{\psi } \dot{\psi } + k_{\psi } \psi = \sum\limits_{i = 1}^{3} {m_{i} } rl_{i} \left[ {\dot{\varphi }_{i}^{2} \sin (\varphi_{i} - \theta_{i} ) - \ddot{\varphi }_{i} \cos (\varphi_{i} - \theta_{i} )} \right] \hfill \\ J_{i} \ddot{\varphi }_{i} + f_{i} \dot{\varphi }_{i} = T_{ei} - T_{Li} ,{\kern 1pt} {\kern 1pt} {\kern 1pt} {\kern 1pt} i = 1,2,3 \hfill \\ \end{gathered}$$

where, *T*_*Li*_ is the torque loads of three motors and it can be derived as Eq. (3).

3 $$T_{Li} = m_{i} r[\ddot{y}\cos \varphi_{i} - \ddot{x}\sin \varphi_{i} + l_{i} \dot{\psi }^{2} \sin (\varphi_{i} - \theta_{i} ) + l_{i} \ddot{\psi }\cos (\varphi_{i} - \theta_{i} )]$$

The item *T*_*e*_ in Eq. (2) is the electromagnetic torque of the inductor motor. Thus, the model of the inductor motor should be established. In this article, the category of the inductor motor is the squirrel-cage motor. According to the feature of this kind of motor, its rotor winding is short circuit, which can be expressed as the mathematical form, $u_{rd} = u_{rq}$. When the motor is at a stable state, $\phi_{rd}$ = constant and $\phi_{rq} = 0$. With the variables $\omega$-$i_{s}$-$\phi_{r}$, the model of inductor motor in the *d*-*q* rotating coordinate system can be represented as Eq. (4) by literature^24^.

4 $$\begin{gathered} L_{ks} di_{sd} /dt = u_{sd} - R_{ks} i_{sd} + R_{r} L_{m} /L_{r}^{2} \phi_{rd} + \omega_{s} L_{ks} i_{sq} \hfill \\ L_{ks} di_{sq} /dt = u_{sq} - R_{ks} i_{sq} - L_{m} /L_{r} \phi_{rd} \omega - \omega_{s} L_{ks} i_{sd} \hfill \\ d\phi_{rd} /dt = 1/T_{r} (L_{m} i_{sd} - \phi_{rd} ) \hfill \\ d{\uptheta }/dt = L_{m} i_{sq} /T_{r} \phi_{rd} + \omega \hfill \\ T_{e} = 3L_{m} \phi_{rd} i_{sq} /2L_{r} \hfill \\ \end{gathered}$$

**Figure 2:**
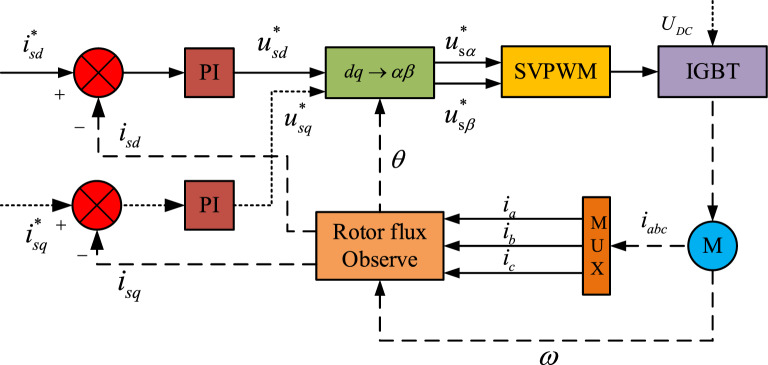
RFOC: rotor flux-oriented control.

The symbols in Eq. (4) are all given in Table 1. *L*_*ks*_ and *R*_*ks*_ respectively represent the leakage inductance and the equivalent resistance of the stator which can be derived as $L_{ks} = L_{s} - L_{m}^{2} /L_{r}$ and $R_{ks} = R_{s} + L_{m}^{2} R_{r} /L_{r}^{2}$. ${\uptheta }$ and $\omega_{s}$ can respectively be represented with the mathematical formulation ${\uptheta } = \int {(\omega + \omega_{s} )} dt$ and $\omega_{s} = L_{m} i_{sq} /\phi_{rd} T_{r}$. To sustain the stability of the motor speed, the rotor flux-oriented control (RFOC) is introduced in the system which is shown in Fig. 2. In the meanwhile, the parameters of three motors are shown in Table 3.

**Table 3:** The parameters of three motors.

Parameters	Motor 1	Motor 2	Motor 3
*P* /kW	0.2	0.2	0.2
*n*_*p*_	3	3	3
*f*_0_ /Hz	50	50	50
*U* /V	220	220	220
*n* /(r/min)	950	950	950
*R*_*s*_ /Ω	40.5	40.5	40.5
*R*_*r*_* /*Ω	12	12	12
*L*_*s*_ /H	1.21275	1.2175	1.21275
*L*_*r*_ /H	1.222	1.225	1.222
*L*_*m*_ /H	1.116	1.116	1.116
$\lambda_{dr}^{*}$/Wb	0.98	0.98	0.98
*f*_1,2,3_ /(Nms/rad)	0.005	0.005	0.005

### The analysis of synchronization and stability of the vibrating system

According to the non-linear dynamical theory, the phase difference between motor 1 and 2 can be expressed as $p\varphi_{1} - q\varphi_{2} = 2\alpha_{1}$, and the phase difference between motor 2 and 3 can be expressed as $q\varphi_{2} - s\varphi_{3} = 2\alpha_{2}$. Set the average phase of three ERs as $\varphi$ which can be represented as $\varphi = (\varphi_{1} + \varphi_{2} + \varphi_{3} )/3$. And then the average speed $\omega_{0}$ in the period *T* can be derived as $\omega_{0} { = }\int_{0}^{T} {\dot{\varphi }{\text{d}}t} /T$. With the small parameter method, the speed and accelerated speed of three ERs are respectively $\dot{\varphi }_{i} = (1{ + }\varepsilon_{i} )\omega_{0}$ and $\ddot{\varphi }_{i} = \dot{\varepsilon }_{i} \omega_{0}$, *i* = 1,2,3. $\varepsilon_{i}$ is the small perturbation parameter. $\omega_{0}$ that is the mean value of the average speed of three motors equals to a constant. Then, the responses in three directions can be derived as Eq. (5)

5 $$\begin{gathered} x = \frac{{ - r_{m} r}}{{\mu_{x} }}\left[ {\sum\limits_{i = 1}^{3} {\eta_{i} } \cos (\varphi_{i} + \gamma_{x} )} \right] \hfill \\ y = \frac{{ - r_{m} r}}{{\mu_{y} }}\left[ {\sum\limits_{i = 1}^{3} {\eta_{i} } \sin (\varphi_{i} + \gamma_{y} )} \right] \hfill \\ \psi = \frac{{ - r_{m} r}}{{\mu_{\psi } l_{e} }}\left[ {\sum\limits_{i = 1}^{3} {\eta_{i} } r_{li} \sin (\varphi_{i} - \theta_{i} + \gamma_{\psi } )} \right] \hfill \\ \end{gathered}$$

where, $$\omega_{x}^{2} = k_{x} /M$$, $$\omega_{y}^{2} = k_{y} /M$$, $$\omega_{\psi }^{2} = k_{\psi } /J$$, $$\xi_{x} = f_{x} /\left( {2\sqrt {k_{x} M} } \right)$$, $$\xi_{y} = f_{y} /\left( {2\sqrt {k_{y} M} } \right)$$, $$\xi_{\psi } = f_{\psi } /\left( {2\sqrt {k_{\psi } J} } \right)$$, $$\tan \gamma_{x} = 2\xi_{x} \omega_{x} /\left( {\mu_{x} \omega_{0} } \right)$$, $$\tan \gamma_{y} = 2\xi_{y} \omega_{y} /\left( {\mu_{y} \omega_{0} } \right)$$, $$\tan \gamma_{\psi } = 2\xi_{\psi } \omega_{\psi } /\left( {\mu_{\psi } \omega_{0} } \right)$$, $$\mu_{x} = 1 - \omega_{x}^{2} /\omega_{0}^{2}$$, $$\mu_{y} = 1 - \omega_{y}^{2} /\omega_{0}^{2}$$, $$\mu_{\psi } = 1 - \omega_{\psi }^{2} /\omega_{0}^{2}$$, $$r_{m} = m_{0} /M$$, $$\eta_{i} = m_{i} /m_{0}$$, $$r_{li} = l_{i} /l_{e}$$.

**Figure 3:**
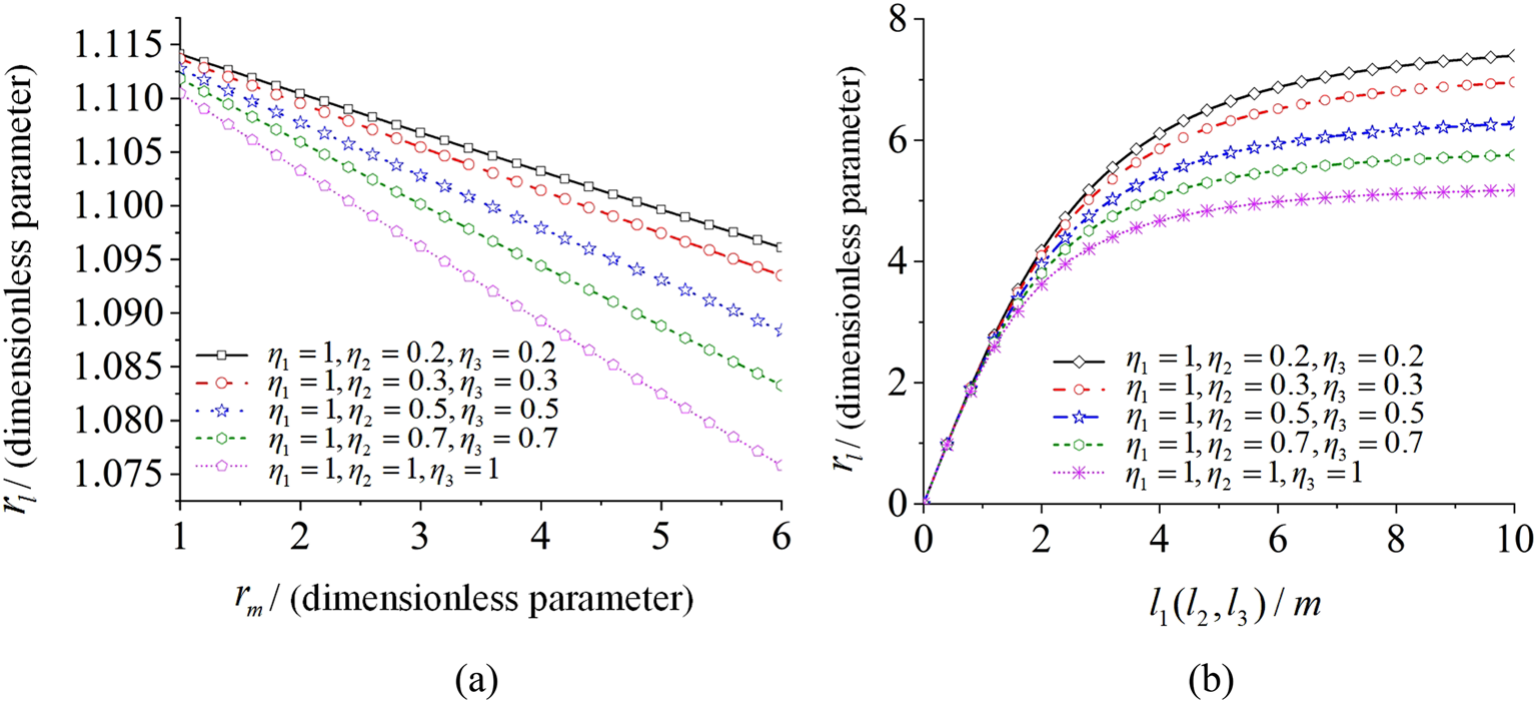
The feature analysis of three parameters.

From the equations above, the feature of the parameters r_*li*_, r_m_, and *l*_*i*_ are analyzed in the situation *l*_1_ = *l*_2_ = *l*_3_. And then, the relationships among the three parameters are illustrated with different parameter $\eta_{i}$ in Fig. 3. The results represent that the closer the masses of three ERs are, the better their synchronous capability is.

Taking the small parameters into Eq. (2), Eq. (6) can be obtained with the integrate method in the $2\pi$ period.

6 $$J_{i} \dot{\overline{\varepsilon }}_{i} \omega_{0} + f_{i} \omega_{0} (1 + \overline{\varepsilon }_{i} ) = \overline{T}_{ei} - \overline{T}_{Li} (i = 1,2,3)$$

where, the torque loads in Eq. (6) can be expressed with small parameters as

7 $$\overline{T}_{Li} = m_{0} r^{2} \omega_{0} \left[ {\sum\limits_{j = 1}^{3} {(a_{ij} \dot{\overline{\varepsilon }}_{j} + b_{ij} \overline{\varepsilon }_{j} ) + \kappa_{i} } } \right](i = 1,2,3)$$

**Figure 4:**
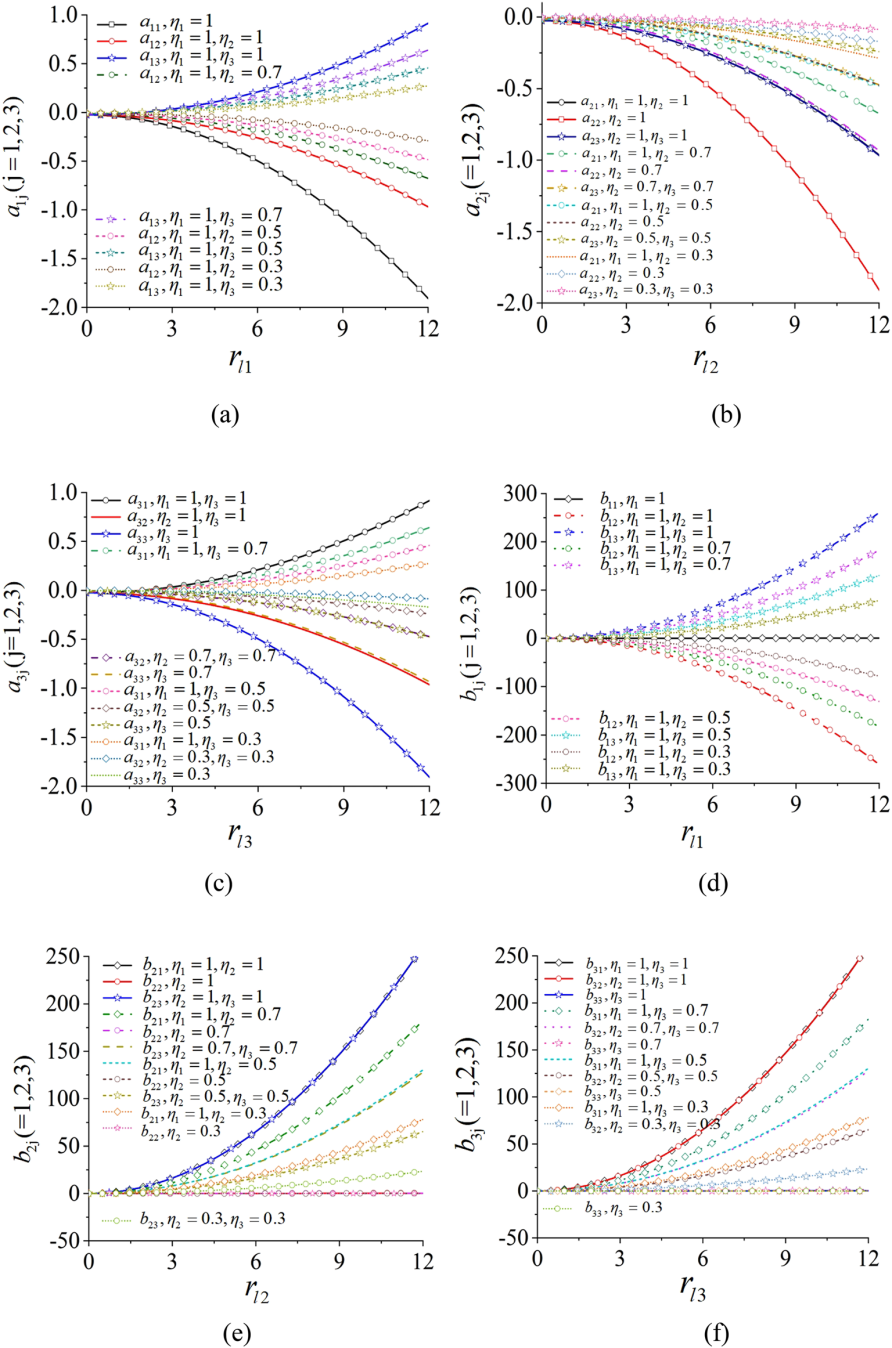
The parameters $a_{ij}$ and $b_{ij}$ with the relationship of $r_{l}$ in the situation $\omega_{0} = 80$ rad/s.

The items $a_{ij}$, $b_{ij}$ and $\kappa_{i}$ which are the second stability coefficient in Eq. (7) are all given in Appendix A. Because $a_{ij}$ and $b_{ij}$ are both associated with r_*li*_, Fig. 4 shows their relationship feature with different parameter $\eta_{i}$. The results indicates that the parameters $a_{ij}$ and $b_{ij}$ are changed with the various parameter $\eta_{i}$ and the coupling dynamical feature of the vibrating system demonstrates the best stable capability in the situation $\eta_{1} = \eta_{2} = \eta_{3}$. By introducing in the small parameter $\varepsilon_{4}$ and $\varepsilon_{5}$, expand the phase differences with the Taylor formula separately and omit the higher order items, $\alpha_{1} = \overline{\alpha }_{1} + \overline{\varepsilon }_{4}$ and $\alpha_{2} = \overline{\alpha }_{2} + \overline{\varepsilon }_{5}$ can be obtained. In the meanwhile, the non-dimensional coupling equations which is numbered as (8) can be derived as

8 $${\mathbf{A}}\mathbf{\dot{\overline{\varepsilon }}} = {\mathbf{B}}\overline{\mathbf{\varepsilon }} + {\varvec{\upupsilon}}$$

where, **A** is a matrix of the moment of inertia and **B** is a stiffness matrix. The symbols in Eq. (8) are listed as below.

$${\mathbf{A}} = \left( {\begin{array}{*{20}c} {a^{\prime}_{11} } & {a^{\prime}_{12} } & {a^{\prime}_{13} } & 0 & 0 \\ {a^{\prime}_{21} } & {a^{\prime}_{22} } & {a^{\prime}_{23} } & 0 & 0 \\ {a^{\prime}_{31} } & {a^{\prime}_{32} } & {a^{\prime}_{33} } & 0 & 0 \\ 0 & 0 & 0 & 1 & 0 \\ 0 & 0 & 0 & 0 & 1 \\ \end{array} } \right),\;{\mathbf{B}} = \left( {\begin{array}{*{20}c} {b^{\prime}_{11} } & {b^{\prime}_{12} } & {b^{\prime}_{13} } & {b^{\prime}_{14} } & {b^{\prime}_{15} } \\ {b^{\prime}_{21} } & {b^{\prime}_{22} } & {b^{\prime}_{23} } & {b^{\prime}_{24} } & {b^{\prime}_{25} } \\ {b^{\prime}_{31} } & {b^{\prime}_{32} } & {b^{\prime}_{33} } & {b^{\prime}_{34} } & {b^{\prime}_{35} } \\ {\omega_{0} /2} & { - \omega_{0} /2} & 0 & 0 & 0 \\ 0 & {\omega_{0} /2} & { - \omega_{0} /2} & 0 & 0 \\ \end{array} } \right),$$

$${\overline{\mathbf{\varepsilon }}} = \left( {\begin{array}{*{20}c} {\overline{\varepsilon }_{1} } & {\overline{\varepsilon }_{2} } & {\overline{\varepsilon }_{3} } & {\overline{\varepsilon }_{4} } & {\overline{\varepsilon }_{5} } \\ \end{array} } \right)^{{\text{T}}}$$, $${\mathbf{\dot{\overline{\varepsilon }}}} = \left( {\begin{array}{*{20}c} {\dot{\overline{\varepsilon }}_{1} } & {\dot{\overline{\varepsilon }}_{2} } & {\dot{\overline{\varepsilon }}_{3} } & {\dot{\overline{\varepsilon }}_{4} } & {\dot{\overline{\varepsilon }}_{5} } \\ \end{array} } \right)^{{\text{T}}}$$, $${{\varvec{\upupsilon}}} = \left( {\begin{array}{*{20}c} {\upsilon_{1} } & {\upsilon_{2} } & {\upsilon_{3} } & 0 & 0 \\ \end{array} } \right)^{{\text{T}}}$$. The items $a^{\prime}_{ij}$, $b^{\prime}_{ij}$ and $\upsilon_{i}$ in Eq. (8) are presented in Appendix B.

**Figure 5:**
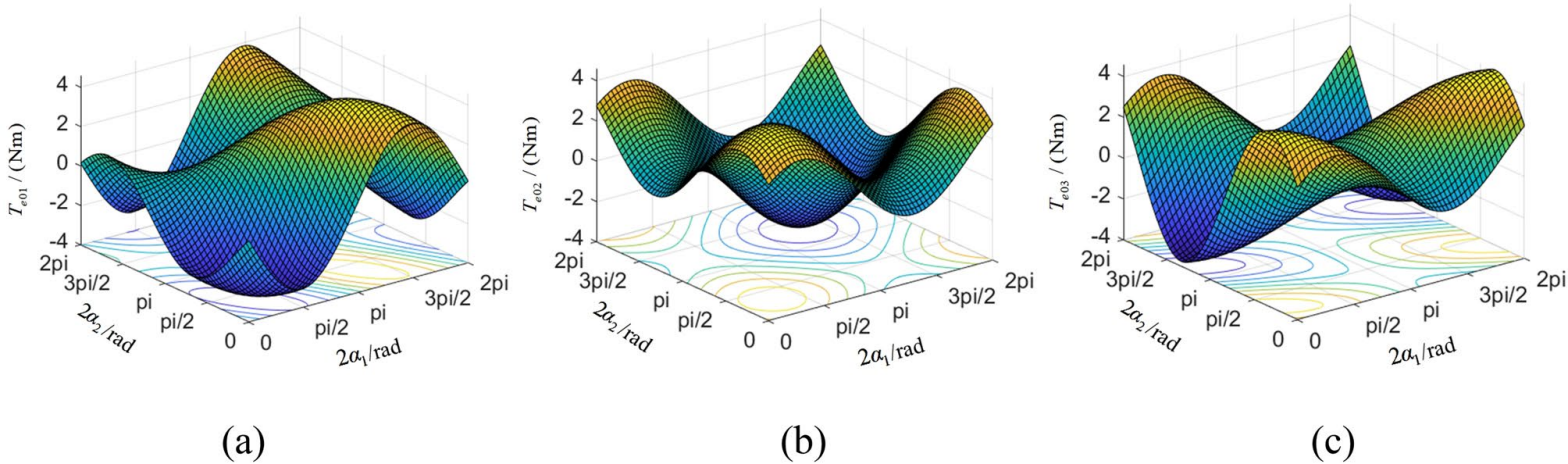
The electromagnetic torque of three motors.

The electromagnetic torque in Eq. (6) can be expressed as

9 $$\overline{T}_{ei} = T_{e0i} - k_{e0i} \overline{\varepsilon }_{i} {\kern 1pt} {\kern 1pt} {\kern 1pt} (i = 1,2,3)$$

where, $T_{e0i} = f_{i} \omega_{0} + m_{i} r^{2} \omega_{0} {\kern 1pt} \kappa_{i} {\kern 1pt} {\kern 1pt} {\kern 1pt} (i = 1,2,3)$ and $k_{e0i}$ can be derived from literature^25^. $T_{e0i}$ is painted as Fig. 5. When the three ERs reach the steady synchronization state, the non-linear parameters $p = q = s = 1$ and the small parameters $\varepsilon_{1} = \varepsilon_{2} = \varepsilon_{3} = 0$, $\dot{\varepsilon }_{1} = \dot{\varepsilon }_{2} = \dot{\varepsilon }_{3} = 0$. The synchronous condition of three ERs can be represented as Eq. (10).

10 $$\left| {T_{e0i} } \right| \le T_{eNi} {\kern 1pt} {\kern 1pt} {\kern 1pt} (i = 1,2,3)$$

where, $T_{eNi} {\kern 1pt} {\kern 1pt} {\kern 1pt} (i = 1,2,3)$ are respectively the rated electromagnetic torque of three motors. When the vibrating system satisfies the synchronization criterion, ${{\varvec{\upupsilon}}} = 0$. Equation (8) can be simplified as

11 $${\mathbf{A}}\user2{\dot{\overline{\varepsilon }}} = {\mathbf{B}}\overline{\user2{\varepsilon }}$$

Because matrix **A** is a non-singular matrix, if the determinant $\left| A \right|$ of matrix A dose not equal to zero, the matrix **A** is an invertible matrix. Thus, Eq. (11) can be expressed as

12 $$\user2{\dot{\overline{\varepsilon }}} = {\mathbf{D}}\overline{\user2{\varepsilon }}$$

where, ${\mathbf{D}} = {\mathbf{A}}^{ - 1} {\mathbf{B}}$. The characteristic equation in Eq. (12) can be obtained by $\det (\lambda {\mathbf{I}} - {\mathbf{D}}) = 0$.

13 $$\lambda^{5} + d_{1} \lambda^{4} + d_{2} \lambda^{3} + d_{3} \lambda^{2} + d_{4} \lambda^{1} + d_{5} = 0$$

$d_{j} \left( {j = 1,2,3,4,5} \right)$ are the coefficient items while $\lambda$ represents the eigenvalue in Eq. (13). The parameters $d_{j} {\kern 1pt} (j = 1,2,3,4,5)$ are given in Appendix C. When the characteristic equation satisfies the Hurwitz conditions in Eq. (14), all the real roots of eigenvalues exist on the left of coordinate (which means to be the negative real roots), the synchronous state of the vibrating system is stable. Otherwise, is unstable.

14 $$\left\{ \begin{gathered} d_{1} > 0, \hfill \\ d_{5} > 0, \hfill \\ d_{1} d_{2} - d_{3} > 0, \hfill \\ d_{1} d_{2} d_{3} - d_{3}^{2} - d_{1}^{2} d_{4} + d_{1} d_{5} > 0, \hfill \\ d_{1} d_{2} d_{3} d_{4} - d_{1}^{2} d_{4}^{2} - d_{1} d_{5} d_{2}^{2} + 2d_{1} d_{4} d_{5} + d_{2} d_{3} d_{5} - d_{3}^{2} d_{4} - d_{5}^{2} > 0 \hfill \\ \end{gathered} \right.$$

## Design and theoretical analysis of the controlling system

### Controller design of the controlling system

**Figure 6:**
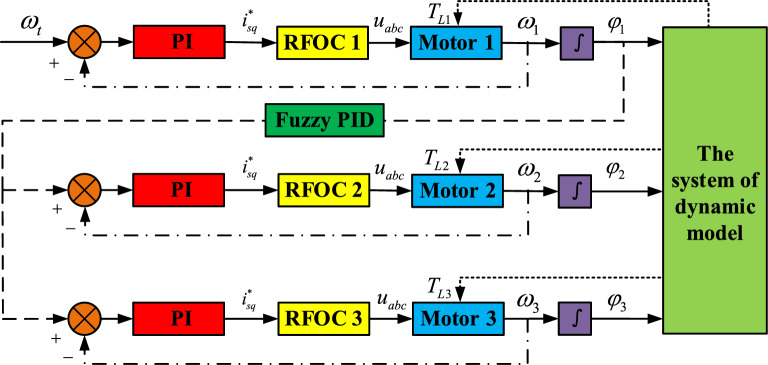
Framework diagram of the controlling system.

In this controlling scheme, a master–slave controlling strategy is introduced in the controlling system as shown in Fig. 6. $\omega_{t}$ as an input target speed is given into the master motor which is named motor 1. The output speed of motor 1 is divided into three functions. One is feedback to the initial speed to promote the accuracy of the motor speed. Another is as an input variable to realize the controlling strategy by the adaptive fuzzy PID method. In the meanwhile, it is converted to the speeds of motor 2 and 3. The other is changed to the phase through an integrate method and then transferred to the dynamical model. The processes of motor 2 and 3 are similar with motor 1’s.

The fuzzy logic model is consisted of two input variables and three output variables. The two input variables are respectively the error *e* and the change rate of error *ec*. The three output variables are respectively the proportionality coefficient $k_{p}$, integral coefficient $k_{I}$ and differential coefficient $k_{D}$. According to the usual fuzzy rules table, the output variables can be obtained through Eqs. (15) with forty-nine rules which are established in this section.

15 $$K_{\iota } = \frac{{\left[ {\sum\limits_{j = 1}^{49} {\mu_{{K_{\iota j} }} (e,ec) \times K_{\iota j} } } \right]}}{{\sum\limits_{j = 1}^{49} {\mu_{{K_{\iota j} }} (e,ec)} }}{\kern 1pt} {\kern 1pt} {\kern 1pt} {\kern 1pt} (\iota = P,I,D)$$

### Theoretical analysis of the controlling system

Because the controlling method is applied in this article, the stability of the controlling method should be analyzed. Choosing the speed as the state variable, it can be expressed as $\omega = \dot{\varphi }$. And then, Eq. (2) can be derived as

16 $$J_{i} \dot{\omega }_{i} + f_{i} \omega_{i} = K_{{T_{i} }} u_{i} - W_{i} {\kern 1pt} {\kern 1pt} {\kern 1pt} (i = 1,2,3)$$

$K_{{T_{i} }} u_{i}$ is introduced in the dynamical equation to replace the electromagnetic torque. Where, $K_{{T_{i} }}$ is represented as the constant of the electromagnetic torque, $K_{Ti} = L_{mi} \phi_{rdi} /L_{ri}$, while $u_{i}$ is considered as the controlling variable $i_{qsi}^{ * }$. $W_{i}$ replaces the load torque which is the uncertain loads in this section, $W_{i} = T_{Li} {\kern 1pt} {\kern 1pt} {\kern 1pt}$. Set *e* as the speed error of the motor and then it can be expressed as the difference between the target speed and the actual speed in Eq. (17).

17 $$e = \omega_{t} - \omega$$

Thus, the tracing error ***E*** can be given as a column vector, ${\mathbf{E}} = [e,\dot{e}]^{{\text{T}}}$. Because $u_{i}$ is the controlling variable, the controlling law is defined as

18 $$u = J/K_{T} \left[ { - \hat{f}(x|\theta_{f} ) + \dot{\omega }_{t} + {\mathbf{K}}^{{\text{T}}} {\mathbf{E}} + (f\omega - W)/J} \right]$$

***K*** is composed of the parameters *k*_*p*_ and *k*_*I*_, ${\mathbf{K}} = [k_{p} ,k_{I} ]^{{\text{T}}}$. The weight coefficient $\theta_{f}$ in the function $\hat{f}(x)$ can be expressed as $\hat{f}(x|\theta_{f} ) = \theta_{f}^{{\text{T}}} {{\varvec{\upxi}}}(x)$. Where, ${{\varvec{\upxi}}}(x)$ is a fuzzy vector. Then the adaptive law of the fuzzy system is represented as

19 $${\dot{\mathbf{\theta }}}_{f} = - \gamma E^{{\text{T}}} {\text{P}}b{{\varvec{\upxi}}}(x)$$

$\gamma$ is a positive constant and **P** is a positive definite matrix.

Taking Eq. (18) into Eq. (16), the dynamical equation with closed loop of the fuzzy controlling system can be expressed as

20 $${\dot{\text{E}}} = {{\varvec{\Lambda}}}{\text{E}} + {\text{b}}\left[ {\hat{f}(x|\theta_{f} ) - f(x)} \right]$$

where, $${{\varvec{\Lambda}}} = \left( {\begin{array}{*{20}c} 0 & 1 \\ { - k_{p} } & { - k_{I} } \\ \end{array} } \right)$$, $${\mathbf{b}} = \left( \begin{gathered} 0 \hfill \\ 1 \hfill \\ \end{gathered} \right)$$.

To guarantee the boundedness of the weight coefficient $\theta_{f}$, the optimal weight coefficient $\theta_{f}^{ * }$ is introduced in the controlling system with a convex set $\Omega_{f}$ which contains $\theta_{f}$, $\theta_{f} \in \Omega_{f}$. Thus, $\theta_{f}^{ * }$ is constructed as

21 $$\theta_{f}^{*} = \arg \mathop {\min }\limits_{{\theta_{f} \in \Omega_{f} }} \left[ {\sup |\hat{f}(x|\theta_{f} ) - f(x)|} \right]$$

Taking Eq. (19) and (21) into Eq. (20), the closed dynamical equation of the fuzzy system can be derived as

22 $$\dot{E} = {{\varvec{\Lambda}}}E + b\left[ {(\theta_{f} - \theta_{f}^{*} )^{{\text{T}}} \xi (x) + \Delta } \right]$$

$\Delta$ is defined as the minimum approximation error and is expressed as $\Delta = \hat{f}(x|\theta_{f}^{*} ) - f(x)$. To accord the adaptive law with the condition of the fuzzy system, the difference between the tracing error*** E*** and the parameter error $\theta_{f} - \theta_{f}^{*}$ should be minimum. So, a Lyapunov function is defined as

23 $$V = E^{{\text{T}}} {\mathbf{P}}E/2 + (\theta_{f} - \theta_{f}^{*} )^{{\text{T}}} (\theta_{f} - \theta_{f}^{*} )/(2\gamma )$$

$\gamma$ is a positive constant. Through introducing a positive definite matrix **Q** with second order, the Lyapunov equation should be satisfied with matrix **P**.

24 $${{\varvec{\Lambda}}}^{{\text{T}}} {\mathbf{P + PA = - Q}}$$

To certify the stability of Lyapunov function, the Lyapunov function in Eq. (23) is divided into two parts. One is $V_{1} = E^{{\text{T}}} {\mathbf{P}}E/2$, the other is ${\mathbf{V}}_{2} = (\theta_{f} - \theta_{f}^{*} )^{{\text{T}}} (\theta_{f} - \theta_{f}^{*} )/(2\gamma )$. According to the Lyapunov criterion, the derivation of ***V***_1_ and ***V***_2_ should be obtained, which are respectively $\dot{V}_{{1}} = - E^{{\text{T}}} {\mathbf{Q}}E/2 + (\theta_{f} - \theta_{f}^{*} )^{{\text{T}}} E^{{\text{T}}} {\mathbf{P}}b\xi (x) + E^{{\text{T}}} {\mathbf{P}}b\Delta$ and ${\dot{\mathbf{V}}}_{2} = (\theta_{f} - \theta_{f}^{*} )^{{\text{T}}} \dot{\theta }_{f} /\gamma$. Thus, the derivate of Eq. (23) can be expressed as ${\dot{\mathbf{V}}} = {\dot{\mathbf{V}}}_{{1}} {\mathbf{ + \dot{V}}}_{{2}} = - {\mathbf{\rm E}}^{{\text{T}}} Q{\mathbf{E}}/2 + {\mathbf{E}}^{{\text{T}}} P{\mathbf{b}}\Delta$. Because of $- {\mathbf{\rm E}}^{{\text{T}}} Q{\mathbf{E}}/2 \le 0$, only if the appropriate parameter $\Delta$ is chosen, ${\dot{\mathbf{V}}} \le 0$ can be obtained. According to LaSalle invariance principle, the controlling system is asymptotic stable. The stability of other motors’ speed error and the stability of phase error can be acquired with the method above.

## Results analysis and discussions

In this section, numerical simulation results corresponding to the dynamical model of Fig. 1 are illustrated. And then, some experiment results are given to verify the simulation results. The feature of vibrating system is discussed.

### Numerical simulation of self-synchronization and controlled synchronization

**Figure 7:**
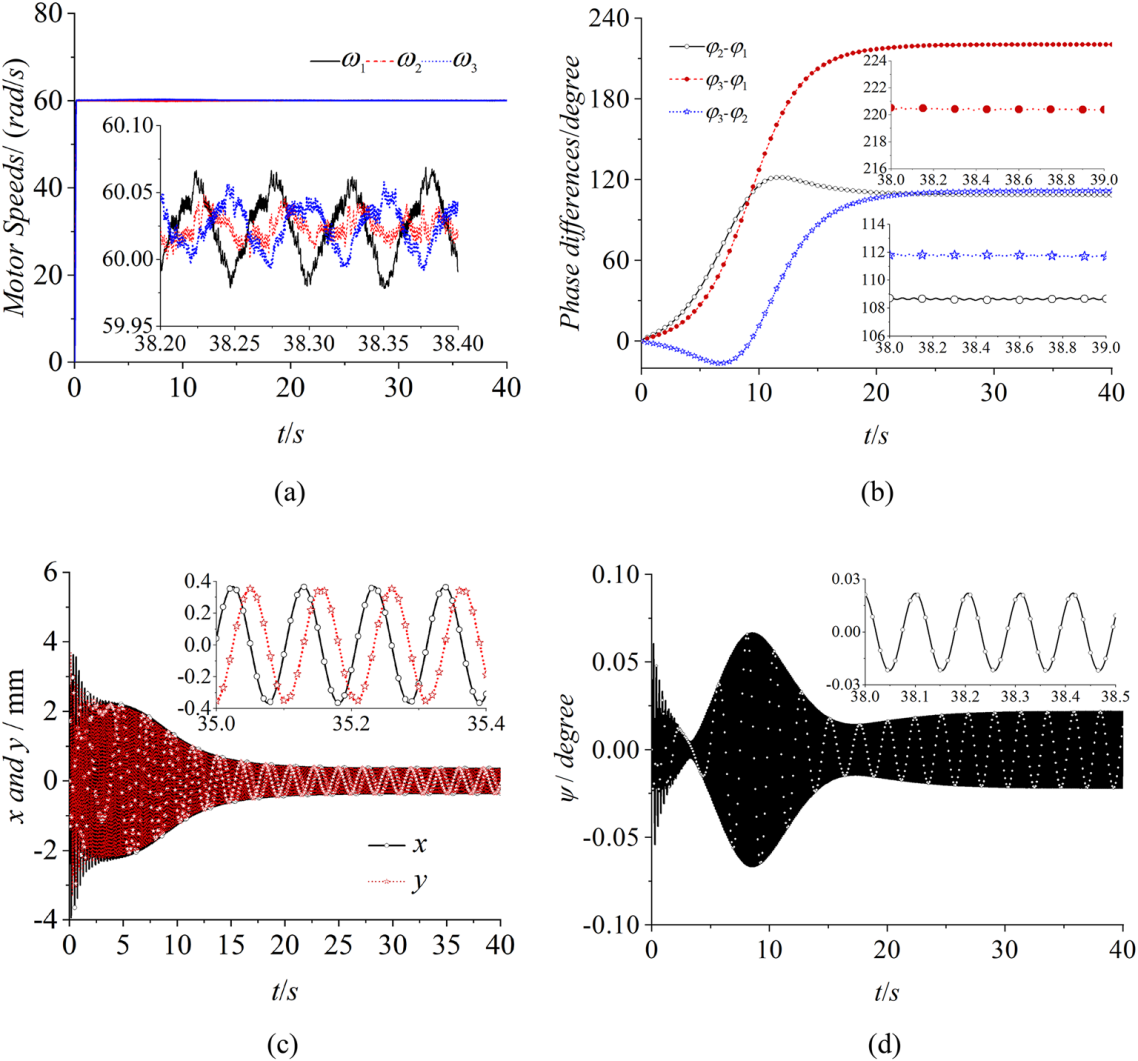
Self-synchronization with three ERs, $\alpha_{0} { = }0$, $\eta_{1} = \eta_{2} = \eta_{3} = 1$.

In Fig. 7, the simulation result of the self-synchronization with three ERs are presented. Figure 7a shows the speed of three motors. With the method of constant voltage frequency ratio, the speeds of three motors fluctuate around 60.02 rad/s which can be realized to reach the synchronous speeds. However, the phase differences between motor 1 and 2 with motor 2 and 3 are separately about 108.5° and 119.5° in Fig. 7b. The result illustrates that the vibrating system obviously can’t reach the synchronous state with zero phase difference which is needed in the engineering. From Fig. 7c, it can be known that the amplitude of the vibrating system is counteracted due to the phase differences in Fig. 7b. Thus, the vibrating system appears an angle of oscillation with 0.02° in Fig. 7d. From the results in Fig. 7, it can be concluded that the vibrating system can’t realize the synchronous state with zero phase difference. And then, the trajectory of the rigid body can’t meet the engineering acquirements.

**Figure 8:**
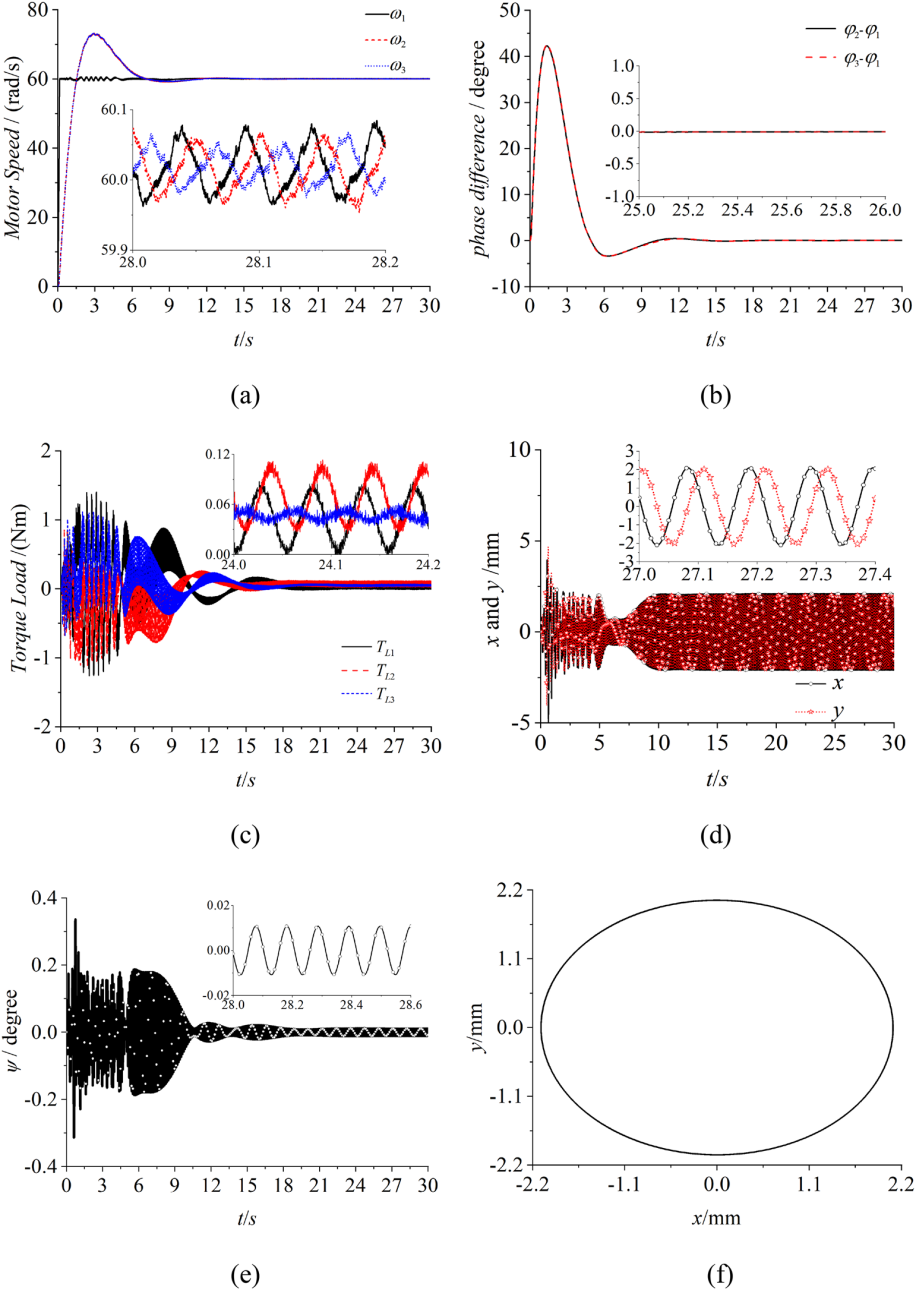
Controlled synchronization with three ERs, $\alpha_{0} { = }0$, $\eta_{1} = \eta_{2} = \eta_{3} = 1$, $l_{1} = l_{2} = 0.32m,l_{3} = 0.3m$, (motor 1 and 2 approach the *y* axis).

**Figure 9:**
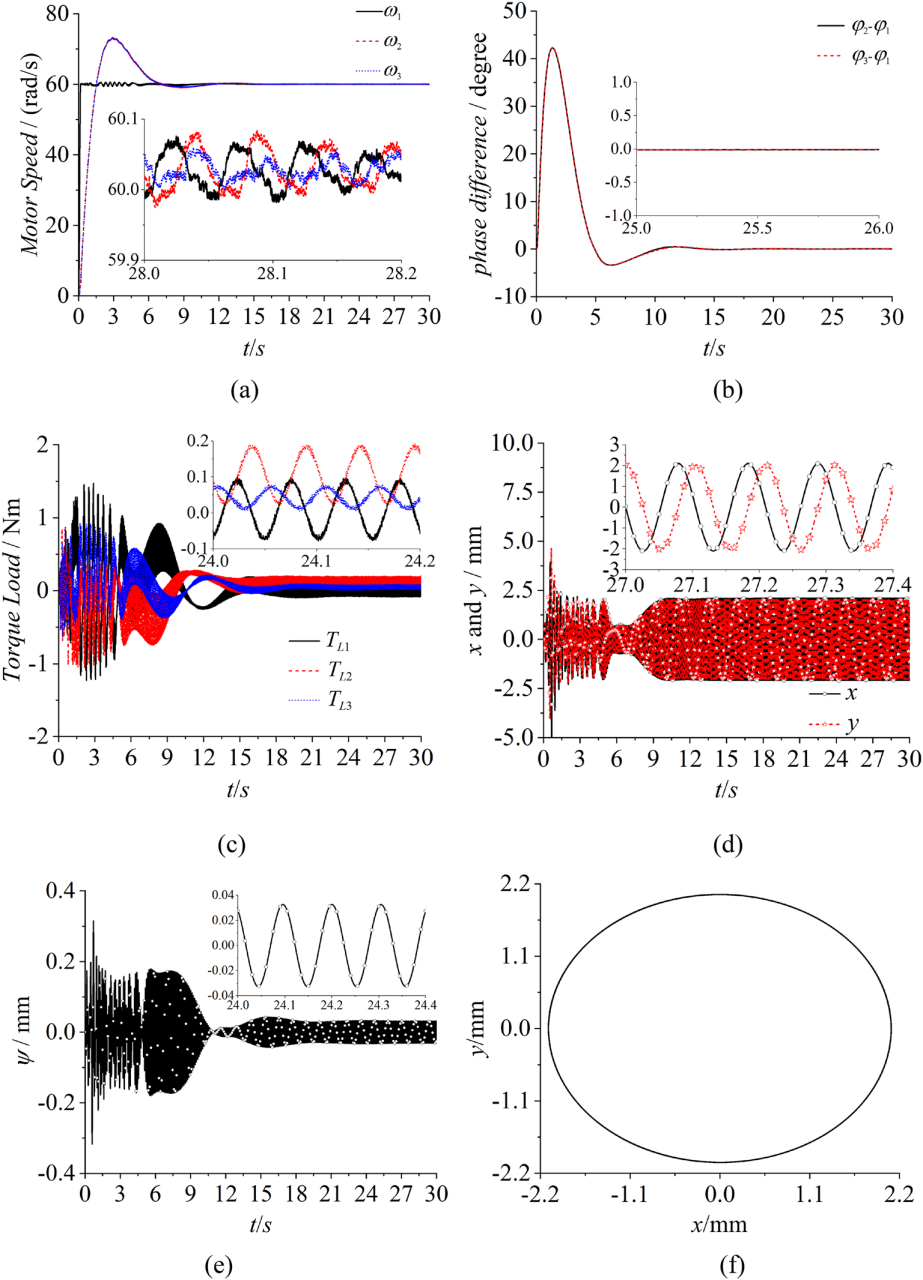
Controlled synchronization with three ERs, $\alpha_{0} { = }0$, $\eta_{1} = \eta_{2} = \eta_{3} = 1$, $l_{1} = l_{2} = 0.45m,l_{3} = 0.3m$, (motor 1 and 2 approach both sides of the rigid body).

To solve this problem, the fuzzy PID method is introduced in the self-synchronization motion to realize the synchronous state with zero phase difference. From Fig. 8a, the speeds of three motors are all about 60 rad/s which equal to the target speed. Figure 8b demonstrates that the phase differences between motor 1 and 2 with motor 1 and 3 are both approximate to 0. And this result represents that the three ERs realize the controlled synchronization motion. In Fig. 8c, values of the torque load are between 0 and 0.12. These values are less than the absolute values of the electromagnetic torque. Thus, the three motor can operate normally and won’t appear the phenomenon of motor blocking. From Fig. 8d,f, the results illustrate that the amplitudes of three ERs are superimposed and the angle of swing of the vibrating system is very small. The vibrating system realizes the controlled synchronization motion and the elliptical trace which is needed in the engineering. To expound the feature of distance between motor 1 and 2, the distance is expanded from center to the sides which is shown in Fig. 9. According to Fig. 9, the speeds and phase differences are both similar with them in Fig. 8. However, the torque loads of three motors enlarge in Fig. 9c due to the increasing of the swing angle in Fig. 9e. The results indicate that although the parameters $l_{1}$ and $l_{2}$ increase with increasing the distance between motor 1 and 2, this change has little influence on the controlled synchronization motion. Because the stable controlling method changes the intrinsic dynamical feature of the vibrating system. Thus, to realize the miniaturization of the vibrating system, the parameters in Fig. 8 are more suitable for the engineering. This is the reason that the vibrating system is in a circular distribution rather than one straight line.

### Experimental verification of self-synchronization and controlled synchronization

**Figure 10:**
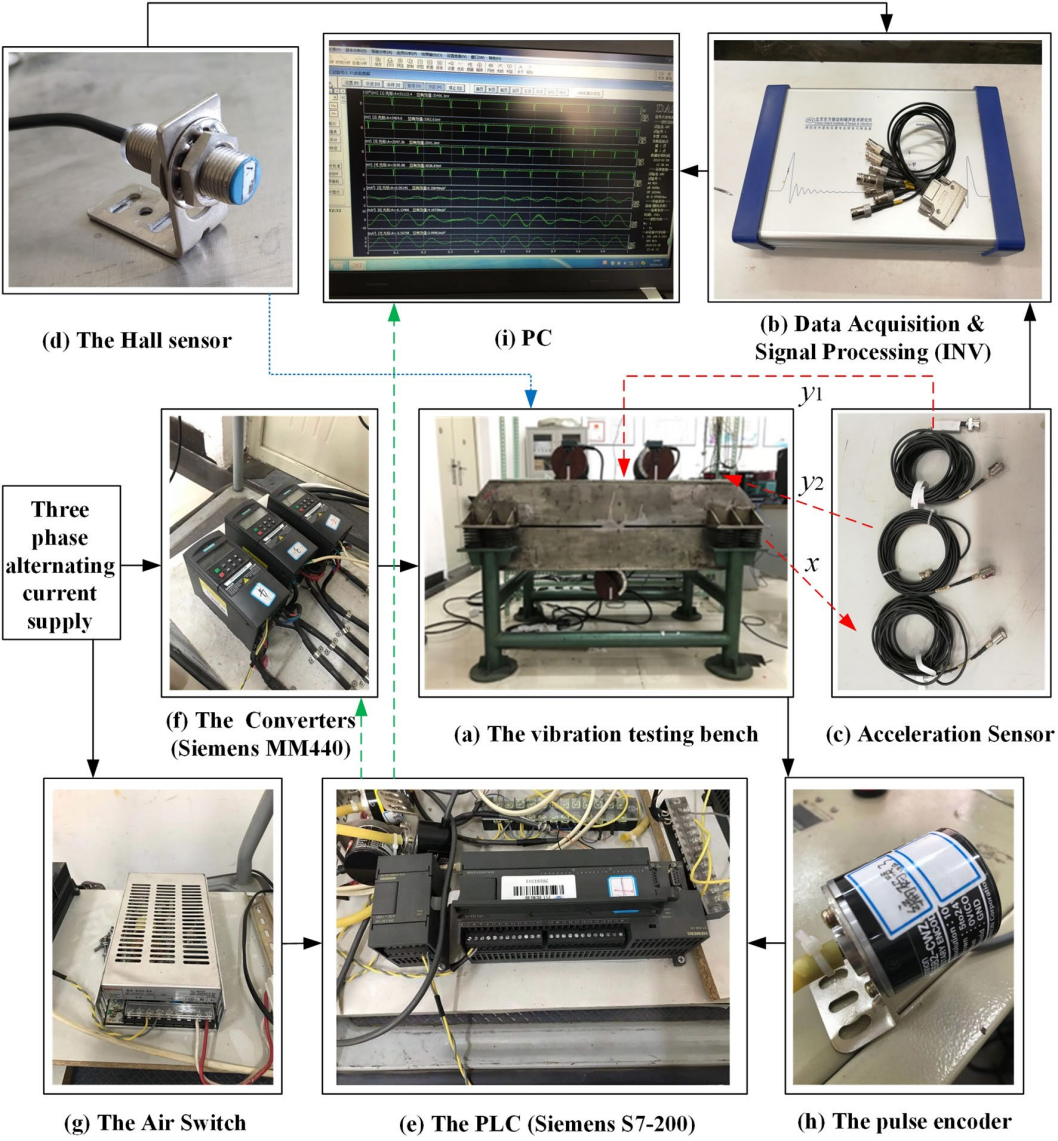
Experimental facilities.

**Figure 11:**
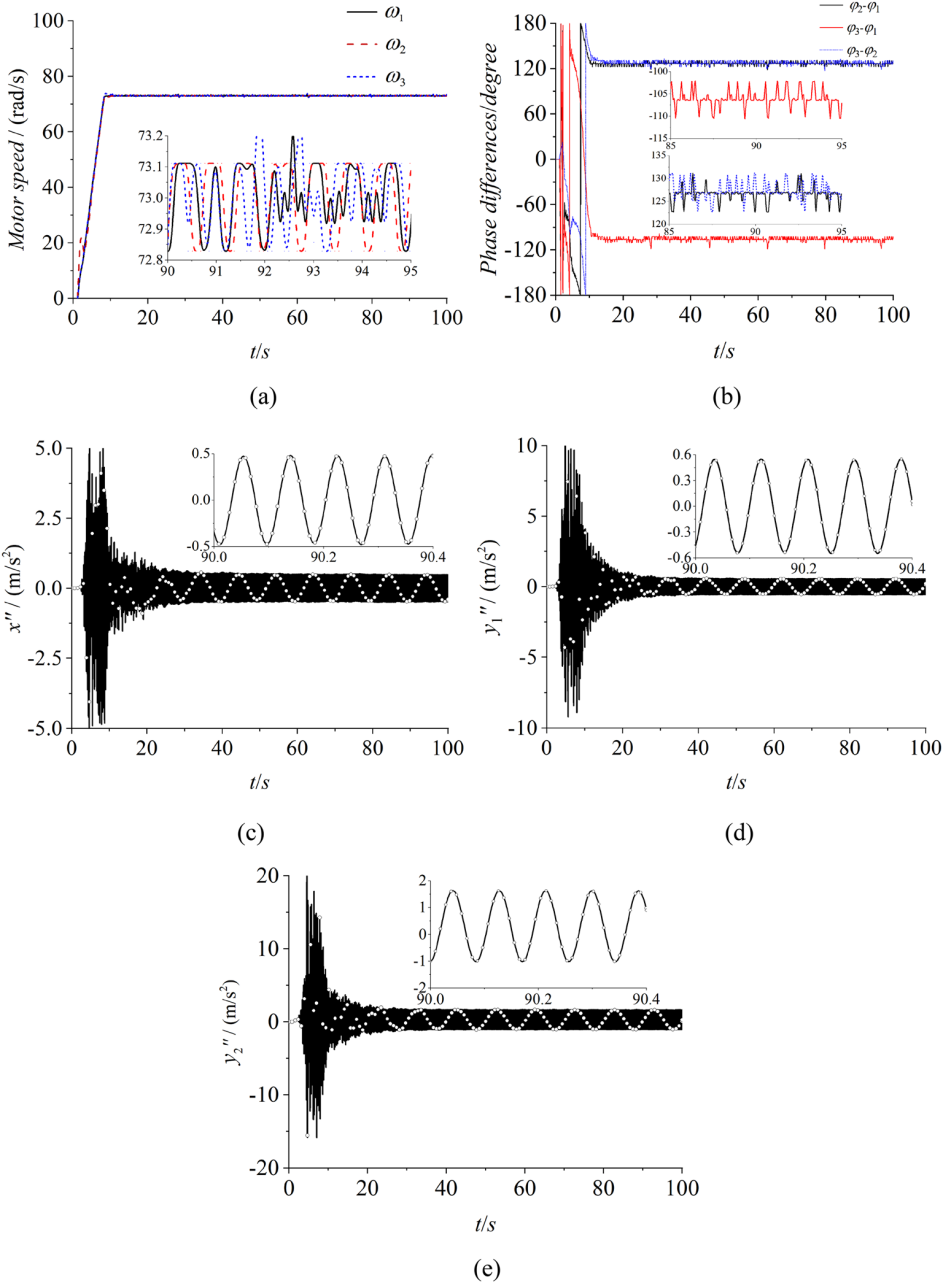
Experiment of self-synchronization with three ERs, $\alpha_{0} { = }0$, $\eta_{1} = \eta_{2} = \eta_{3} = 1$.

**Figure 12:**
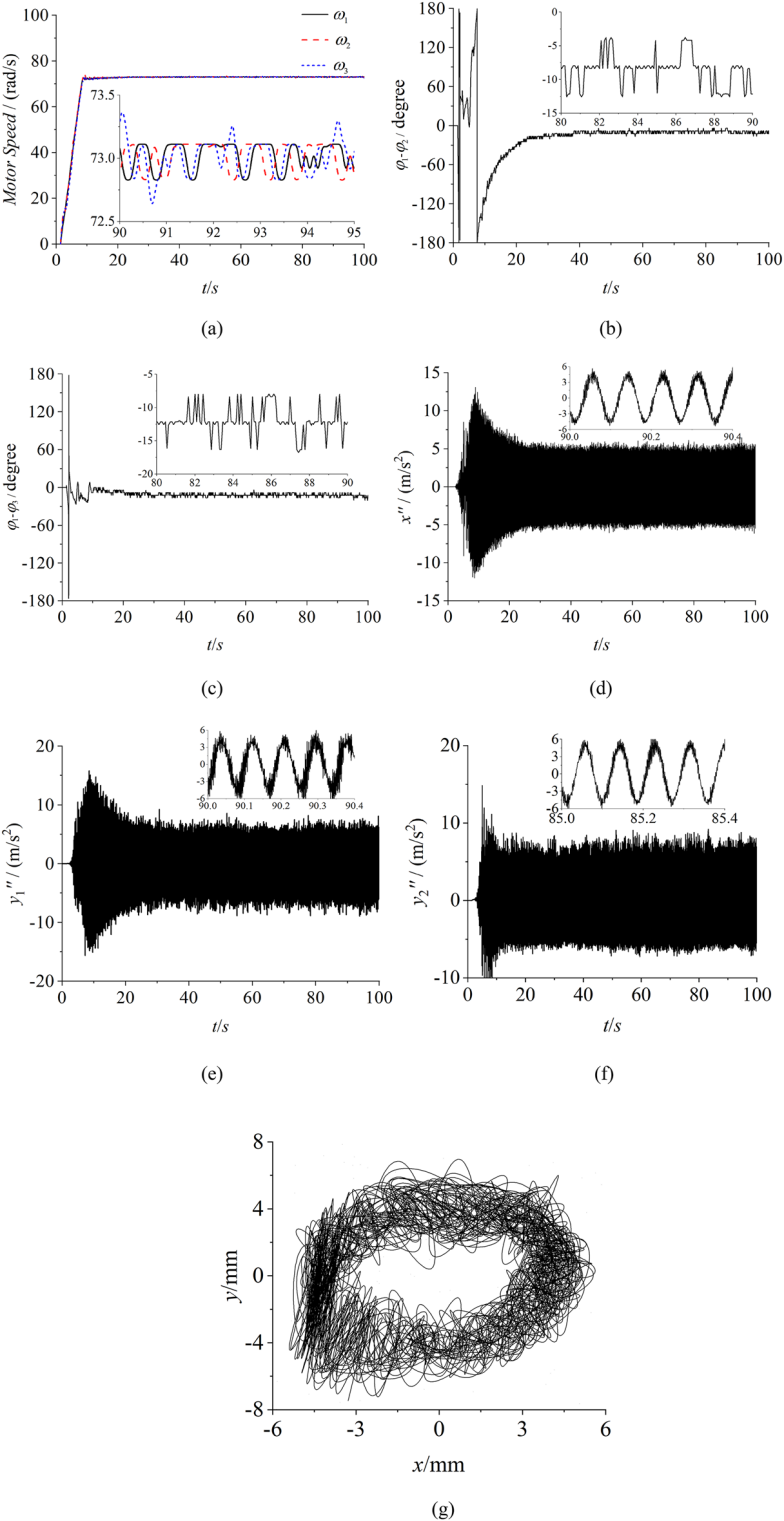
Experiment of controlled synchronization with three ERs, $\alpha_{0} { = }0$, $\eta_{1} = \eta_{2} = \eta_{3} = 1$ (motor 1 and 2 approach the *y* axis). (**a**) speeds, (**b**) phase difference between motor 1 and 2, (**c**) phase differences between motor 1 and 3, (**d**) response in the x direction, (**e**) response in the y1 direction, (**f**) response in the y2 direction, (**g**) The motion trace of the rigid body .

**Figure 13:**
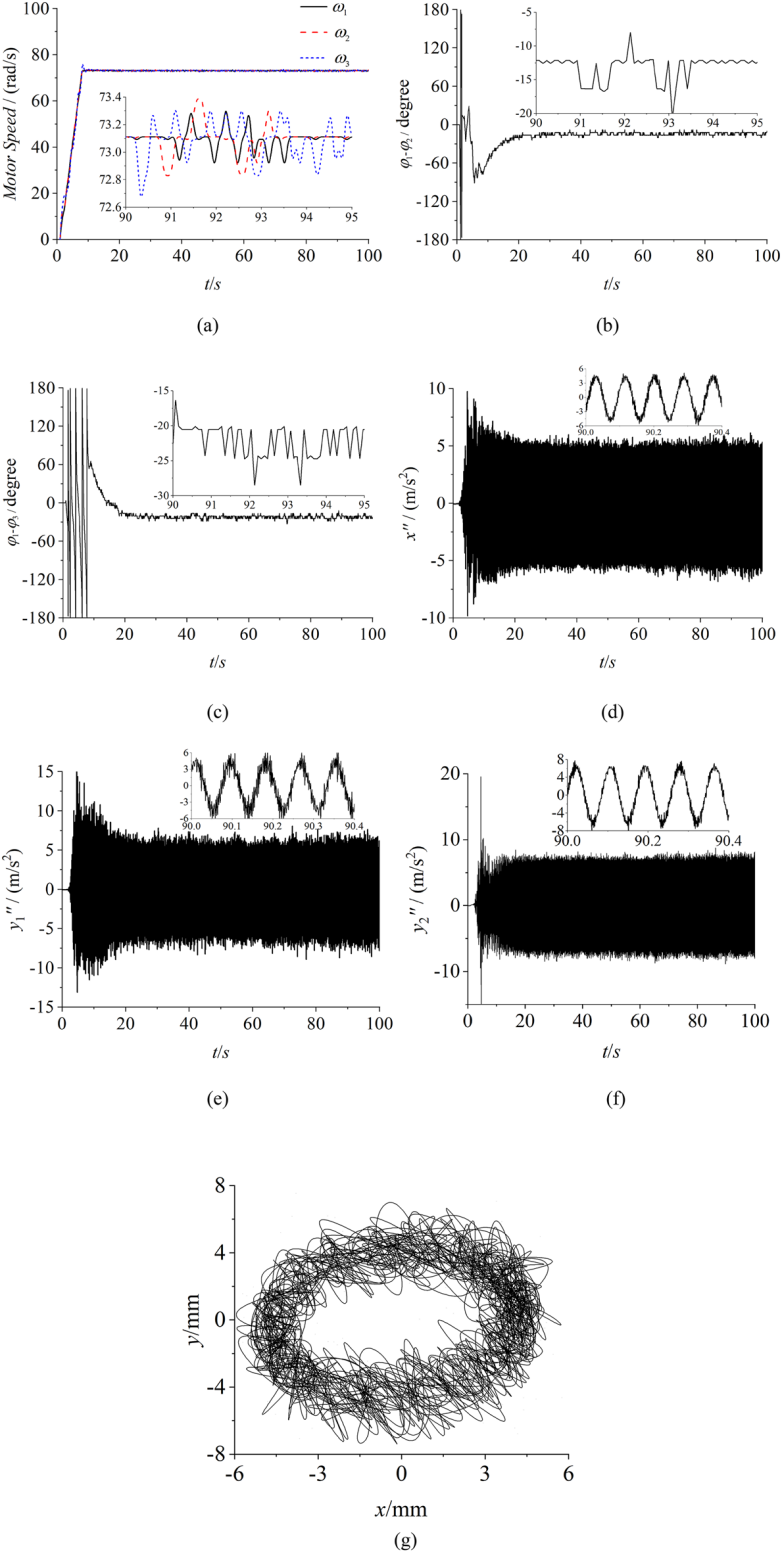
Experiment of controlled synchronization with three ERs, $\alpha_{0} { = }0$, $\eta_{1} = \eta_{2} = \eta_{3} = 1$ (motor 1 and 2 approach both sides of the rigid body). (**a**) speeds, (**b**) phase difference between motor 1 and 2, (**c**) phase differences between motor 1 and 3, (**d**) response in the x direction, (**e**) response in the y1 direction, (**f**) response in the y2 direction, (**g**) The motion trace of the rigid body

To certify the correctness of the proposed theory and the consistency with numerical simulation, some experiments of self-synchronization and controlled synchronization are given. The experimental facilities are firstly listed in Fig. 10 which illuminates the experimental procedure in the meanwhile. In this experiment, the frequencies of three inductor motors are respectively set as 35 Hz through three convertors. Through the calculation conversion, the speeds of three motors can be calculated as 73.2 rad/s which can be recognized as the theorical value. In Fig. 11a, the speeds of three motors all have a large fluctuation around 73 rad/s. Due to the existence of the error in the experiment, this result can be recognized to realize the synchronous speed. In Fig. 11b, the phase difference between motor 1 and 2 is about 127°, and this result is similar with the result in Fig. 7b. The same result can be obtained from the phase difference between motor 1 and 3. From Fig. 11c,e, the results indicates that the amplitude responses of three directions are all counteracted. Thus, the experimental result is consistence with the numerical simulation result of the self-synchronization. Figures 12 and 13 both represent the experimental results of the controlled synchronization motion and are respectively responses to the simulation results in Figs. 8 and 9. With the controlling method, the speeds of three motors exist less fluctuation. The phase differences can be considered small enough to realize the controlled synchronization motion. As shown from (d) to (g) in Figs. 12 and 13, the responses amplitudes in three directions are all in superposition state and the values are approximately the same. This result adequately represents that the vibrating system can realize the elliptic motion trace. Therefore, the experimental results are consistent with the numerical results and the effectiveness and correctness of the theorical method are verified.

## Conclusions

This article investigates controlled synchronization of three co-rotating exciters based on a circular distribution in a vibratory system. Through the theorical feature analysis, the results indicate that the self-synchronization motion is depended the parameter of $\eta_{i}$ and $l_{i}$. When $l_{i}$ can’t meet the condition of zero phase difference in the self-synchronization motion, the ellipse motion trace in the vibrating system can’t be realized. Therefore, the stability of the vibrating system depends on the controlling method and suitable controlling strategy in the controlled synchronization motion which can realize the ellipse motion trace in the vibrating system. Compared with the former researches, this article adopts a circle distribution in the dynamical model. It can further reduce the structure size of the vibrating system, which is the purpose of using the controlled synchronization instead of the self-synchronization. In the meanwhile, an adaptive fuzzy PID method is introduced. Because this method can be independent to the dynamical model and the parameter PID don’t need to be founded with lots of time compared with the former methods.

### Supplementary Information

Supplementary Information.

## Data Availability

The datasets generated during the current study are available from the corresponding/first author on reasonable request.
