# Supplementary material for: Controlled synchronization of three co-rotating exciters based on a circular distribution in a vibratory system
Source: Sci Rep. 2024 Feb 29;14:5026. doi: 10.1038/s41598-024-55680-8 (PMC10904827; doi:10.1038/s41598-024-55680-8)
Supplement: Supplementary file 1 — Supplementary Information. [file 41598_2024_55680_MOESM1_ESM.pdf]

Controlled synchronization of three co-rotating exciters based on a circular distribution in a vibratory system

Lei Jia[1]*, Yang Tian[2], Ziliang Liu[1], Xin Zhang[1]

[1]School of Mechanical Engineering, Shenyang Ligong University, Shenyang 110159, China.

[2]School of Mechanical Engineering, Liaoning Engineering Vocational College, Tieling 112008, China.

*Corresponding author. Email: jialeizsq@126.com

Appendix A

The coefficient items in equation (7).

$$a_{11} = -\eta_1^2[r_m \cos\gamma_x / \mu_x + r_m \cos\gamma_y / \mu_y + r_m r_{l1}^2 \cos\gamma_\psi / \mu_\psi]/2 \tag{A.1}$$

$$a_{12} = -\eta_1\eta_2[r_m \cos(2\alpha_1 - \gamma_x)/\mu_x + r_m \cos(2\alpha_1 - \gamma_y)/\mu_y + r_m r_{l1} r_{l2} \cos(2\alpha_1 + \theta_2 - \theta_1 - \gamma_\psi)/\mu_\psi]/2 \tag{A.2}$$

$$a_{13} = -\eta_1\eta_3[r_m \cos(2\alpha_1 + 2\alpha_2 - \gamma_x)/\mu_x + r_m \cos(2\alpha_1 + 2\alpha_2 - \gamma_y)/\mu_y + r_m r_{l1} r_{l3} \cos(2\alpha_1 + 2\alpha_2 + \theta_3 - \theta_1 - \gamma_\psi)/\mu_\psi]/2 \tag{A.3}$$

$$a_{21} = -\eta_1\eta_2[r_m \cos(2\alpha_1 + \gamma_x)/\mu_x + r_m \cos(2\alpha_1 + \gamma_y)/\mu_y + r_m r_{l1} r_{l2} \cos(2\alpha_1 - \theta_1 + \theta_2 + \gamma_\psi)/\mu_\psi]/2 \tag{A.4}$$

$$a_{22} = -\eta_2^2[\mathrm{r}_m \cos\gamma_x / \mu_x + \mathrm{r}_m \cos\gamma_y / \mu_y + \mathrm{r}_m r_{l2}^2 \cos\gamma_\psi / \mu_\psi]/2 \tag{A.5}$$

$$a_{23} = -\eta_2\eta_3[r_m \cos(2\alpha_2 - \gamma_x)/\mu_x + r_m \cos(2\alpha_2 - \gamma_y)/\mu_y + r_m r_{l2} r_{l3} \cos(2\alpha_2 + \theta_3 - \theta_2 - \gamma_\psi)/\mu_\psi]/2 \tag{A.6}$$

$$a_{31} = -\eta_1\eta_3[r_m \cos(2\alpha_1 + 2\alpha_2 + \gamma_x)/\mu_x + r_m \cos(2\alpha_1 + 2\alpha_2 + \gamma_y)/\mu_y + r_m r_{l1} r_{l3} \cos(2\alpha_1 + 2\alpha_2 - \theta_1 + \theta_3 + \gamma_\psi)/\mu_\psi]/2 \tag{A.7}$$

$$a_{32} = -\eta_2\eta_3[r_m \cos(2\alpha_2 + \gamma_x)/\mu_x + r_m \cos(2\alpha_2 + \gamma_y)/\mu_y + r_m r_{l2} r_{l3} \cos(2\alpha_2 - \theta_2 + \theta_3 + \gamma_\psi)/\mu_\psi]/2 \tag{A.8}$$

$$a_{33} = -\eta_3^2[r_m \cos\gamma_x / \mu_x + r_m \cos\gamma_y / \mu_y + r_m r_{l3}^2 \cos\gamma_\psi / \mu_\psi]/2 \tag{A.9}$$

$$b_{11} = \eta_1^2\omega_0[r_m \sin\gamma_x / \mu_x + r_m \sin\gamma_y / \mu_y + r_m r_{l1}^2 \sin\gamma_\psi / \mu_\psi] \tag{A.10}$$

$$b_{12} = -\eta_1\eta_2\omega_0[r_m \sin(2\alpha_1 - \gamma_x)/\mu_x + r_m \sin(2\alpha_1 - \gamma_y)/\mu_y + r_m r_{l1} r_{l2} \sin(2\alpha_1 + \theta_2 - \theta_1 - \gamma_\psi)/\mu_\psi] \tag{A.11}$$

$$b_{13} = -\eta_1\eta_3\omega_0[r_m \sin(2\alpha_1 + 2\alpha_2 - \gamma_x)/\mu_x + r_m \sin(2\alpha_1 + 2\alpha_2 - \gamma_y)/\mu_y + r_m r_{l1} r_{l3} \sin(2\alpha_1 + 2\alpha_2 + \theta_3 - \theta_1 - \gamma_\psi)/\mu_\psi] \tag{A.12}$$

$$b_{14} = -\eta_1\eta_2\omega_0[r_m \cos(2\alpha_1 - \gamma_x)/\mu_x + r_m \cos(2\alpha_1 - \gamma_y)/\mu_y + r_m r_{l1} r_{l2} \cos(2\alpha_1 + \theta_2 - \theta_1 - \gamma_\psi)]/\mu_\psi - \eta_1\eta_3\omega_0[r_m \cos(2\alpha_1 + 2\alpha_2 - \gamma_x)/\mu_x + r_m \cos(2\alpha_1 + 2\alpha_2 - \gamma_y)/\mu_y + r_m r_{l1} r_{l3} \cos(2\alpha_1 + 2\alpha_2 + \theta_3 - \theta_1 - \gamma_\psi)/\mu_\psi] \tag{A.13}$$

$$b_{15} = -\eta_1\eta_3\omega_0[r_m \cos(2\alpha_1 + 2\alpha_2 - \gamma_x)/\mu_x + r_m \cos(2\alpha_1 + 2\alpha_2 - \gamma_y)/\mu_y + r_m r_{l1} r_{l3} \cos(2\alpha_1 + 2\alpha_2 + \theta_3 - \theta_1 - \gamma_\psi)/\mu_\psi] \tag{A.14}$$

$$b_{21} = \eta_1\eta_2\omega_0[r_m \sin(2\alpha_1+\gamma_x)/\mu_x + r_m \sin(2\alpha_1+\gamma_y)/\mu_y + r_m r_{l1} r_{l2} \sin(2\alpha_1-\theta_1+\theta_2+\gamma_\psi)/\mu_\psi] \quad \text{(A.15)}$$

$$b_{22} = \eta_2^2\omega_0[r_m \sin\gamma_x/\mu_x + r_m \sin\gamma_y/\mu_y + r_m r_{l2}^2 \sin\gamma_\psi/\mu_\psi] \quad \text{(A.16)}$$

$$\begin{aligned} b_{23} = -\eta_2\eta_3\omega_0[&r_m \sin(2\alpha_2-\gamma_x)/\mu_x + r_m \sin(2\alpha_2-\gamma_y)/\mu_y \\ &+ r_m r_{l2} r_{l3} \sin(2\alpha_2+\theta_3-\theta_2-\gamma_\psi)/\mu_\psi] \end{aligned} \quad \text{(A.17)}$$

$$\begin{aligned} b_{24} = \eta_1\eta_2\omega_0[&r_m \cos(2\alpha_1+\gamma_x)/\mu_x + r_m \cos(2\alpha_1+\gamma_y)/\mu_y \\ &+ r_m r_{l1} r_{l2} \cos(2\alpha_1-\theta_1+\theta_2+\gamma_\psi)/\mu_\psi] \end{aligned} \quad \text{(A.18)}$$

$$\begin{aligned} b_{25} = -\eta_2\eta_3\omega_0[&r_m \cos(2\alpha_2-\gamma_x)/\mu_x + r_m \cos(2\alpha_2-\gamma_y)/\mu_y \\ &+ r_m r_{l2} r_{l3} \cos(2\alpha_2+\theta_3-\theta_2-\gamma_\psi)/\mu_\psi] \end{aligned} \quad \text{(A.19)}$$

$$\begin{aligned} b_{31} = \eta_1\eta_3\omega_0[&r_m \sin(2\alpha_1+2\alpha_2+\gamma_x)/\mu_x + r_m \sin(2\alpha_1+2\alpha_2+\gamma_y)/\mu_y \\ &+ r_m r_{l1} r_{l3} \sin(2\alpha_1+2\alpha_2-\theta_1+\theta_3+\gamma_\psi)/\mu_\psi] \end{aligned} \quad \text{(A.20)}$$

$$\begin{aligned} b_{32} = \eta_2\eta_3\omega_0[&r_m \sin(2\alpha_2+\gamma_x)/\mu_x + r_m \sin(2\alpha_2+\gamma_y)/\mu_y \\ &+ r_m r_{l2} r_{l3} \sin(2\alpha_2-\theta_2+\theta_3+\gamma_\psi)/\mu_\psi] \end{aligned} \quad \text{(A.21)}$$

$$b_{33} = \eta_3^2\omega_0[r_m \sin\gamma_x/\mu_x + r_m \sin\gamma_y/\mu_y + r_m r_{l3}^2 \sin\gamma_\psi/\mu_\psi] \quad \text{(A.22)}$$

$$\begin{aligned} b_{34} = \eta_1\eta_3\omega_0[&r_m \cos(2\alpha_1+2\alpha_2+\gamma_x)/\mu_x + r_m \cos(2\alpha_1+2\alpha_2+\gamma_y)/\mu_y \\ &+ r_m r_{l1} r_{l3} \cos(2\alpha_1+2\alpha_2-\theta_1+\theta_3+\gamma_\psi)/\mu_\psi] \end{aligned} \quad \text{(A.23)}$$

$$\begin{aligned} b_{35} = \eta_1\eta_3\omega_0[&r_m \cos(2\alpha_1+2\alpha_2+\gamma_x)/\mu_x + r_m \cos(2\alpha_1+2\alpha_2+\gamma_y)/\mu_y \\ &+ r_m r_{l1} r_{l3} \cos(2\alpha_1+2\alpha_2-\theta_1+\theta_3+\gamma_\psi)/\mu_\psi] + \eta_2\eta_3\omega_0[r_m \cos(2\alpha_2+\gamma_x)/\mu_x \\ &+ r_m \cos(2\alpha_2+\gamma_y)/\mu_y + r_m r_{l2} r_{l3} \cos(2\alpha_2-\theta_2+\theta_3+\gamma_\psi)/\mu_\psi] \end{aligned} \quad \text{(A.24)}$$

$$\kappa_i = (\sum_{j=1}^{3} b_{ij})/2 \ (i=1,2,3) \quad \text{(A.25)}$$

Appendix B

$$a'_{11} = \eta_1 + a_{11},\ a'_{12} = a_{12},\ a'_{13} = a_{13} \quad \text{(B.1)}$$

$$a'_{21} = a_{21},\ a'_{22} = \eta_2 + a_{22},\ a'_{23} = a_{23} \quad \text{(B.2)}$$

$$a'_{31} = a_{31},\ a'_{32} = a_{32},\ a'_{33} = \eta_3 + a_{33} \quad \text{(B.3)}$$

$$b'_{11} = -[f_1/(m_0 r^2) + k_{e01}/(m_0 r^2\omega_0) + b_{11}],\ b'_{12} = -b_{12},\ b'_{13} = -b_{13},\ b'_{14} = -b_{14},\ b'_{15} = -b_{15} \quad \text{(B.4)}$$

$$b'_{21} = -b_{21},\ b'_{22} = -[f_2/(m_0 r^2) + k_{e02}/(m_0 r^2\omega_0) + b_{22}],\ b'_{23} = -b_{23},\ b'_{24} = -b_{24},\ b'_{25} = -b_{25} \quad \text{(B.5)}$$

$$b'_{31}=-b_{31}\,,\ b'_{32}=-b_{32}\,,\ b'_{33}=-[f_3/(m_0r^2)+k_{e03}/(m_0r^2\omega_0)+b_{33}],\ b'_{34}=-b_{34}\,,\ b'_{35}=-b_{35} \quad \text{(B.6)}$$

$$\upsilon_i=T_{e0i}/(m_0r^2\omega_0)-f_i/(m_0r^2)-\kappa_i\,(i=1,2,3) \quad \text{(B.7)}$$

Appendix C

$$d_j=D_j/D_0\,(j=1,2,3,4,5) \quad \text{(C.1)}$$

$$D_0=4a'_{11}a'_{22}a'_{33}-4a'_{11}a'_{23}a'_{32}-4a'_{12}a'_{21}a'_{33}+4a'_{12}a'_{23}a'_{31}+4a'_{13}a'_{21}a'_{32}-4a'_{13}a'_{22}a'_{31} \quad \text{(C.2)}$$

$$\begin{aligned}D_1=&-4a'_{11}a'_{22}b'_{33}-4a'_{11}a'_{23}b'_{32}-4a'_{11}a'_{32}b'_{23}-4a'_{11}a'_{33}b'_{22}+4a'_{12}a'_{21}b'_{33}+4a'_{12}a'_{23}b'_{31}\\&+4a'_{12}a'_{31}b'_{23}-4a'_{12}a'_{33}b'_{21}+4a'_{13}a'_{21}b'_{32}-4a'_{13}a'_{22}b'_{31}+4a'_{13}a'_{31}b'_{22}+4a'_{13}a'_{32}b'_{21}\\&+4a'_{21}a'_{32}b'_{13}-4a'_{21}a'_{33}b'_{12}-4a'_{22}a'_{31}b'_{13}-4a'_{22}a'_{33}b'_{11}+4a'_{23}a'_{31}b_{12}+4a'_{23}a'_{32}b'_{11}\end{aligned} \quad \text{(C.3)}$$

$$\begin{aligned}D_2=&\,\omega_0(-2a'_{11}a'_{22}b'_{35}+2a'_{11}a'_{23}b'_{34}-2a'_{11}a'_{23}b'_{35}+2a'_{11}a'_{32}b'_{25}-2a'_{11}a'_{33}b'_{24}\\&+2a'_{11}a'_{33}b'_{25}+2a'_{12}a'_{21}b'_{35}+2a'_{12}a'_{23}b'_{34}-2a'_{12}a'_{31}b'_{25}-2a'_{12}a'_{33}b'_{24}\\&-2a'_{13}a'_{21}b'_{34}+2a'_{13}a'_{21}b'_{35}-2a'_{13}a'_{22}b'_{34}+2a'_{13}a'_{31}b'_{24}-2a'_{13}a'_{31}b'_{25}\\&+2a'_{13}a'_{32}b'_{24}-2a'_{21}a'_{32}b'_{15}+2a'_{21}a'_{33}b'_{14}-2a'_{21}a'_{33}b'_{15}+2a'_{22}a'_{31}b'_{15}\\&+2a'_{22}a'_{33}b'_{14}-2a'_{23}a'_{31}b'_{14}+2a'_{23}a'_{31}b'_{15}-2a'_{23}a'_{32}b'_{14})+4a'_{11}b'_{22}b'_{33}\\&-4a'_{11}b'_{23}b'_{32}+4a'_{12}b'_{21}b'_{33}+4a'_{12}b'_{23}b'_{31}+4a'_{13}b'_{21}b'_{32}+4a'_{13}b'_{22}b'_{31}+4a'_{21}b'_{12}b'_{33}\\&+4a'_{21}b'_{13}b'_{32}+4a'_{22}b'_{11}b'_{33}-4a'_{22}b'_{13}b'_{31}+4a'_{23}b'_{11}b'_{32}+4a'_{23}b'_{12}b'_{31}+4a'_{31}b'_{12}b'_{23}\\&+4a'_{31}b'_{13}b'_{22}+4a'_{32}b'_{11}b'_{23}+4a'_{32}b'_{13}b'_{21}+4a'_{33}b'_{11}b'_{22}-4a'_{33}b'_{12}b'_{21}\end{aligned} \quad \text{(C.4)}$$

$$\begin{aligned}D_3=&\,\omega_0(2a'_{11}b'_{22}b'_{35}+2a'_{11}b'_{23}b'_{34}-2a'_{11}b'_{23}b'_{35}+2a'_{11}b'_{24}b'_{33}+2a'_{11}b'_{25}b'_{32}\\&-2a'_{11}b'_{25}b'_{33}+2a'_{12}b'_{21}b'_{35}+2a'_{12}b'_{23}b'_{34}+2a'_{12}b'_{24}b'_{33}-2a'_{12}b'_{25}b'_{31}\\&-2a'_{13}b'_{21}b'_{34}+2a'_{13}b'_{21}b'_{35}+2a'_{13}b'_{22}b'_{34}+2a'_{13}b'_{24}b'_{31}+2a'_{13}b'_{24}b'_{32}\\&-2a'_{13}b'_{25}b'_{31}+2a'_{21}b'_{12}b'_{35}-2a'_{21}b'_{13}b'_{34}+2a'_{21}b'_{13}b'_{35}-2a'_{21}b'_{14}b'_{33}\\&-2a'_{21}b'_{15}b'_{32}+2a'_{21}b'_{15}b'_{33}+2a'_{22}b'_{11}b'_{35}-2a'_{22}b'_{13}b_{34}'-2a'_{22}b'_{14}b'_{33}\\&+2a'_{22}b'_{15}b'_{31}-2a'_{23}b'_{11}b'_{34}+2a'_{23}b'_{11}b'_{35}+2a'_{23}b'_{12}b'_{34}-2a'_{23}b'_{14}b'_{31}\\&-2a'_{23}b'_{14}b'_{32}+2a'_{23}b'_{15}b'_{31}-2a'_{31}b'_{12}b'_{25}+2a'_{31}b'_{13}b'_{24}-2a'_{31}b'_{13}b'_{25}\\&-2a'_{31}b'_{14}b'_{23}-2a'_{31}b'_{15}b'_{22}+2a'_{31}b'_{15}b'_{23}-2a'_{32}b'_{11}b'_{25}+2a'_{32}b'_{13}b'_{24}\\&-2a'_{32}b'_{14}b'_{23}-2a'_{32}b'_{15}b'_{21}+2'a_{33}b'_{11}b'_{24}-2a'_{33}b'_{11}b'_{25}-2a'_{33}b'_{12}b'_{24}\\&+2a'_{33}b'_{14}b'_{21}-2a'_{33}b'_{14}b'_{22}-2a'_{33}b'_{15}b'_{21})-4b'_{11}b'_{22}b'_{33}+4b'_{11}b'_{23}b'_{32}\\&+4b'_{12}b'_{21}b'_{33}+4b'_{12}b'_{23}b'_{31}+4b'_{13}b'_{21}b'_{32}+4b'_{13}b'_{22}b'_{31}\end{aligned} \quad \text{(C.5)}$$

$$
\begin{aligned}
D_4 = {\omega_0}^2 (& a'_{11} b'_{24} b'_{35} - a'_{11} b'_{25} b'_{34} + a'_{12} b'_{24} b'_{35} - a'_{12} b'_{25} b'_{34} + a'_{13} b'_{24} b'_{35} - a'_{13} b'_{25} b'_{34} \\
& - a'_{21} b'_{14} b'_{35} + a'_{21} b'_{15} b'_{34} - a'_{22} b'_{14} b'_{35} + a'_{22} b'_{15} b'_{34} - a'_{23} b'_{14} b'_{35} \\
& + a'_{23} b'_{15} b'_{34} + a'_{31} b'_{14} b'_{25} - a'_{31} b'_{15} b'_{24} + a'_{32} b'_{14} b'_{25} - a'_{32} b'_{15} b'_{24} \\
& + a'_{33} b'_{14} b'_{25} - a'_{33} b'_{15} b'_{24}) - \omega_0 (2b'_{11} b'_{22} b'_{35} - 2b'_{11} b'_{23} b'_{34} + 2b'_{11} b'_{23} b'_{35} \\
& - 2b'_{11} b'_{24} b'_{33} - 2b'_{11} b'_{25} b'_{32} + 2b'_{11} b'_{25} b'_{33} + 2b'_{12} b'_{21} b'_{35} + 2b'_{12} b'_{23} b'_{34} \\
& + 2b'_{12} b'_{24} b'_{33} - 2b'_{12} b'_{25} b'_{31} - 2b'_{13} b'_{21} b'_{34} + 2b'_{13} b'_{21} b'_{35} + 2b'_{13} b'_{22} b'_{34} \\
& + 2b'_{13} b'_{24} b'_{31} + 2b'_{13} b'_{24} b'_{32} - 2b'_{13} b'_{25} b'_{31} - 2b'_{14} b'_{21} b'_{33} + 2b'_{14} b'_{22} b'_{33} \\
& - 2b'_{14} b'_{23} b'_{31} - 2b'_{14} b'_{23} b'_{32} - 2b'_{15} b'_{21} b'_{32} + 2b'_{15} b'_{21} b'_{33} - 2b'_{15} b'_{22} b'_{31} \\
& + 2b'_{15} b'_{23} b'_{31})
\end{aligned} \tag{C.6}
$$

$$
\begin{aligned}
D_5 = (& -b'_{11} b'_{24} b'_{35} + b'_{11} b'_{25} b'_{34} + b'_{12} b'_{24} b'_{35} - b'_{12} b'_{25} b'_{34} + b'_{13} b'_{24} b'_{35} \\
& - b'_{13} b'_{25} b'_{34} - b'_{14} b'_{21} b'_{35} + b'_{14} b'_{22} b'_{35} - b'_{14} b'_{23} b'_{35} + b'_{14} b'_{25} b'_{31} \\
& + b'_{14} b'_{25} b'_{32} - b'_{14} b'_{25} b'_{33} + b'_{15} b'_{21} b'_{34} - b'_{15} b'_{22} b'_{34} + b'_{15} b'_{23} b'_{34} \\
& - b'_{15} b'_{24} b'_{31} - b'_{15} b'_{24} b'_{32} + b'_{15} b'_{24} b'_{33}) \omega_0^2
\end{aligned} \tag{C.7}
$$
